# Supplementary material for: Insertion sequence transposition inactivates CRISPR-Cas immunity
Source: Nat Commun. 2023 Jul 20;14:4366. doi: 10.1038/s41467-023-39964-7 (PMC10359306; doi:10.1038/s41467-023-39964-7)
Supplement: Supplementary file 1 — Supplementary Information [file 41467_2023_39964_MOESM1_ESM.pdf]

Supplementary Information

Insertion sequence transposition inactivates CRISPR-Cas immunity

Yong Sheng,[1,4] Hengyu Wang,[1,4] Yixin Ou,[1,2] Yingying Wu,[1,3] Wei Ding,[1] Meifeng Tao,[1,2] Shuangjun Lin,[1,2] Zixin Deng,[1,2,*] Linquan Bai,[1,*] and Qianjin Kang[1,2,*]

[1]State Key Laboratory of Microbial Metabolism, Joint International Research Laboratory of Metabolic & Developmental Sciences, and School of Life Sciences & Biotechnology, Shanghai Jiao Tong University, Shanghai 200240, China.

[2]Haihe Laboratory of Synthetic Biology, Tianjin, 300308, China

[3]National Engineering Research Center of Edible Fungi, Key Laboratory of Applied Mycological Resources and Utilization (South), Ministry of Agriculture and Rural Affairs, Institute of Edible Fungi, Shanghai Academy of Agricultural Sciences, Shanghai 201403, PR China.

[4]These authors contributed equally

[*]Corresponding authors. E-mails: qjkang@sjtu.edu.cn (Q.J.K.),

bailq@sjtu.edu.cn (L.Q.B.) and zxdeng@sjtu.edu.cn (Z.X.D.)

Supplementary Figures

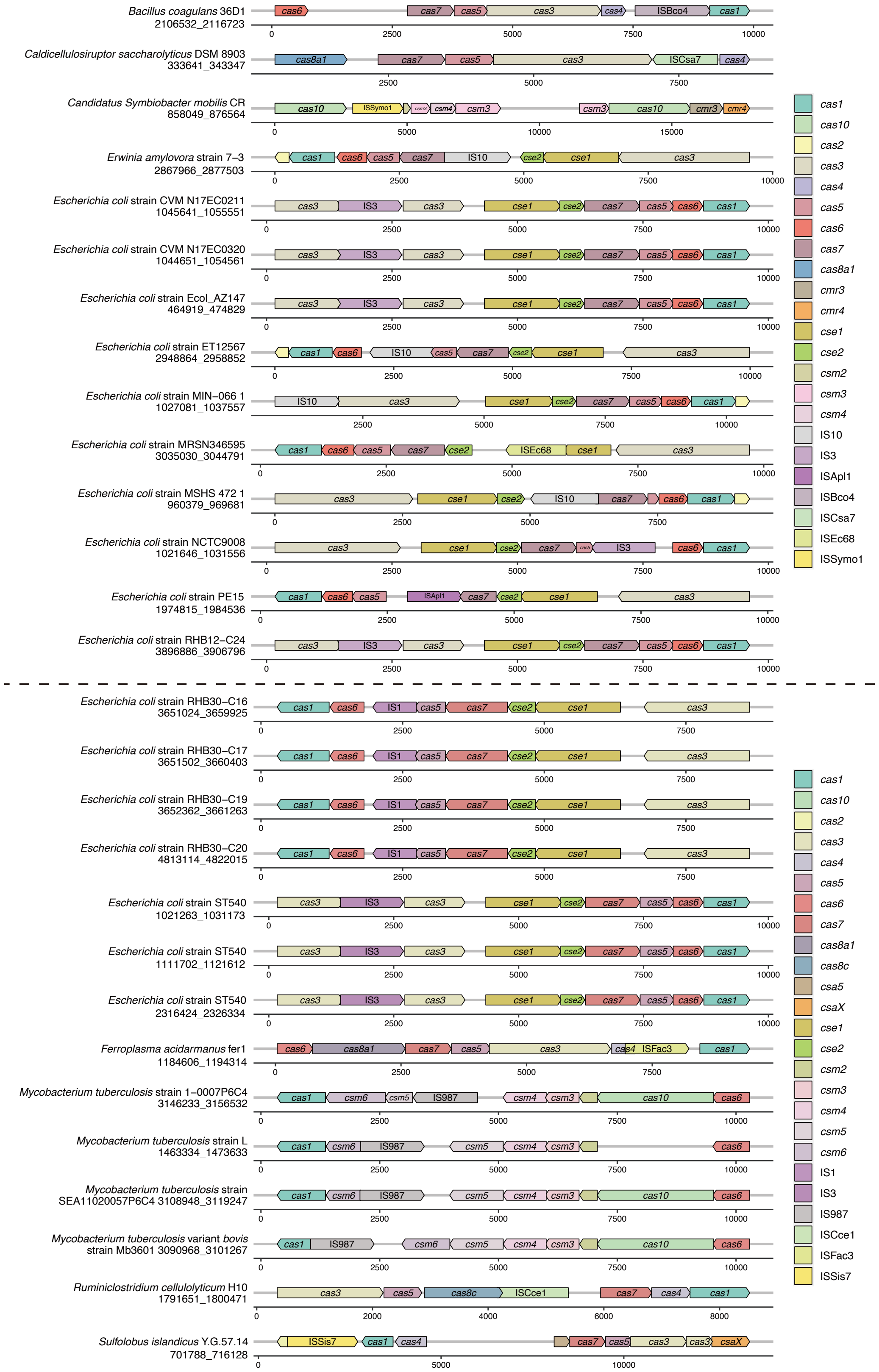

Figure S1. Identification of naturally occurring transpositions of ISs into *cas* genes based on ISfinder database. All 28 identified transposition events are shown above.

Each IS element in the figure represents a unique IS type.

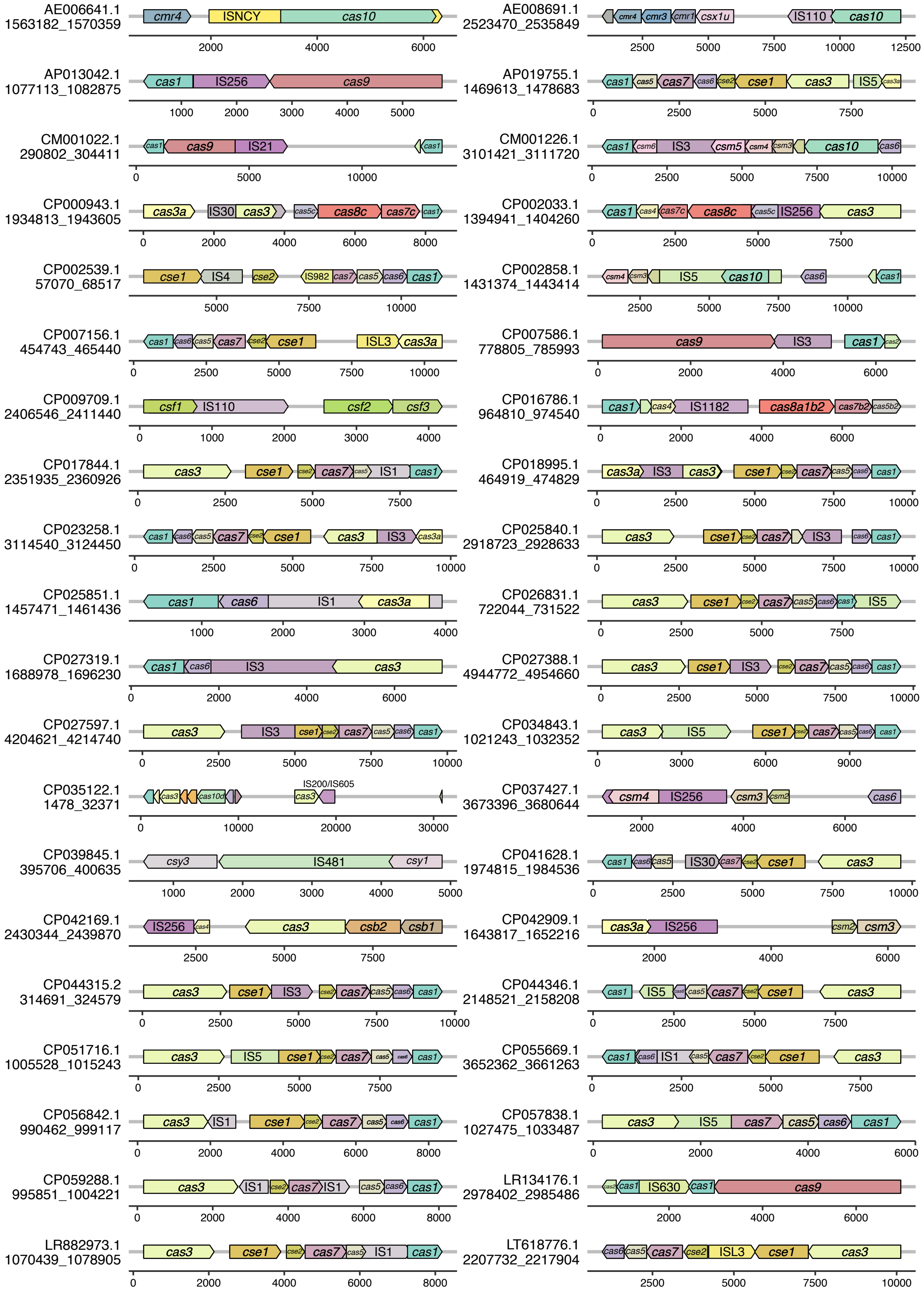
AE006641.1
1563182_1570359
cmr4 ISNCY cas10
AE008691.1
2523470_2535849
cmr4 cmr3 cmr1 csx1u IS110 cas10
AP013042.1
1077113_1082875
cas1 IS256 cas9
AP019755.1
1469613_1478683
cas1 cas5 cas7 cas6 cse2 cse1 cas3 IS5 cas3a
CM001022.1
290802_304411
cas1 cas9 IS21 cas1
CM001226.1
3101421_3111720
cas1 csm6 IS3 csm5 csm4 csm3 cas10 cas6
CP000943.1
1934813_1943605
cas3a IS30 cas3 cas5c cas8c cas7c cas1
CP002033.1
1394941_1404260
cas1 cas4 cas7c cas8c cas5c IS256 cas3
CP002539.1
57070_68517
cse1 IS4 cse2 IS982 cas7 cas5 cas6 cas1
CP002858.1
1431374_1443414
csm4 csm3 IS5 cas10 cas6 cas1
CP007156.1
454743_465440
cas1 cas6 cas5 cas7 cse2 cse1 ISL3 cas3a
CP007586.1
778805_785993
cas9 IS3 cas1 cas2
CP009709.1
2406546_2411440
csf1 IS110 csf2 csf3
CP016786.1
964810_974540
cas1 cas4 IS1182 cas8a1b2 cas7b2 cas5b2
CP017844.1
2351935_2360926
cas3 cse1 cse2 cas7 cas5 IS1 cas1
CP018995.1
464919_474829
cas3a IS3 cas3 cse1 cse2 cas7 cas5 cas6 cas1
CP023258.1
3114540_3124450
cas1 cas6 cas5 cas7 cse2 cse1 cas3 IS3 cas3a
CP025840.1
2918723_2928633
cas3 cse1 cse2 cas7 IS3 cas6 cas1
CP025851.1
1457471_1461436
cas1 cas6 IS1 cas3a
CP026831.1
722044_731522
cas3 cse1 cse2 cas7 cas5 cas6 cas1 IS5
CP027319.1
1688978_1696230
cas1 cas6 IS3 cas3
CP027388.1
4944772_4954660
cas3 cse1 IS3 cse2 cas7 cas5 cas6 cas1
CP027597.1
4204621_4214740
cas3 IS3 cse1 cse2 cas7 cas5 cas6 cas1
CP034843.1
1021243_1032352
cas3 IS5 cse1 cse2 cas7 cas5 cas6 cas1
CP035122.1
1478_32371
cas3 cas10d IS200/IS605 cas3
CP037427.1
3673396_3680644
csm4 IS256 csm3 csm2 cas6
CP039845.1
395706_400635
csy3 IS481 csy1
CP041628.1
1974815_1984536
cas1 cas6 cas5 IS30 cas7 cse2 cse1 cas3
CP042169.1
2430344_2439870
IS256 cas4 cas3 csb2 csb1
CP042909.1
1643817_1652216
cas3a IS256 csm2 csm3
CP044315.2
314691_324579
cas3 cse1 IS3 cse2 cas7 cas5 cas6 cas1
CP044346.1
2148521_2158208
cas1 IS5 cas5 cas7 cse2 cse1 cas3
CP051716.1
1005528_1015243
cas3 IS5 cse1 cse2 cas7 cas5 cas6 cas1
CP055669.1
3652362_3661263
cas1 cas6 IS1 cas5 cas7 cse2 cse1 cas3
CP056842.1
990462_999117
cas3 IS1 cse1 cse2 cas7 cas5 cas6 cas1
CP057838.1
1027475_1033487
cas3 IS5 cas7 cas5 cas6 cas1
CP059288.1
995851_1004221
cas3 IS1 cse2 cas7 IS1 cas5 cas6 cas1
LR134176.1
2978402_2985486
cas2 cas1 IS630 cas1 cas9
LR882973.1
1070439_1078905
cas3 cse1 cse2 cas7 cas5 IS1 cas1
LT618776.1
2207732_2217904
cas6 cas5 cas7 cse2 ISL3 cse1 cas3

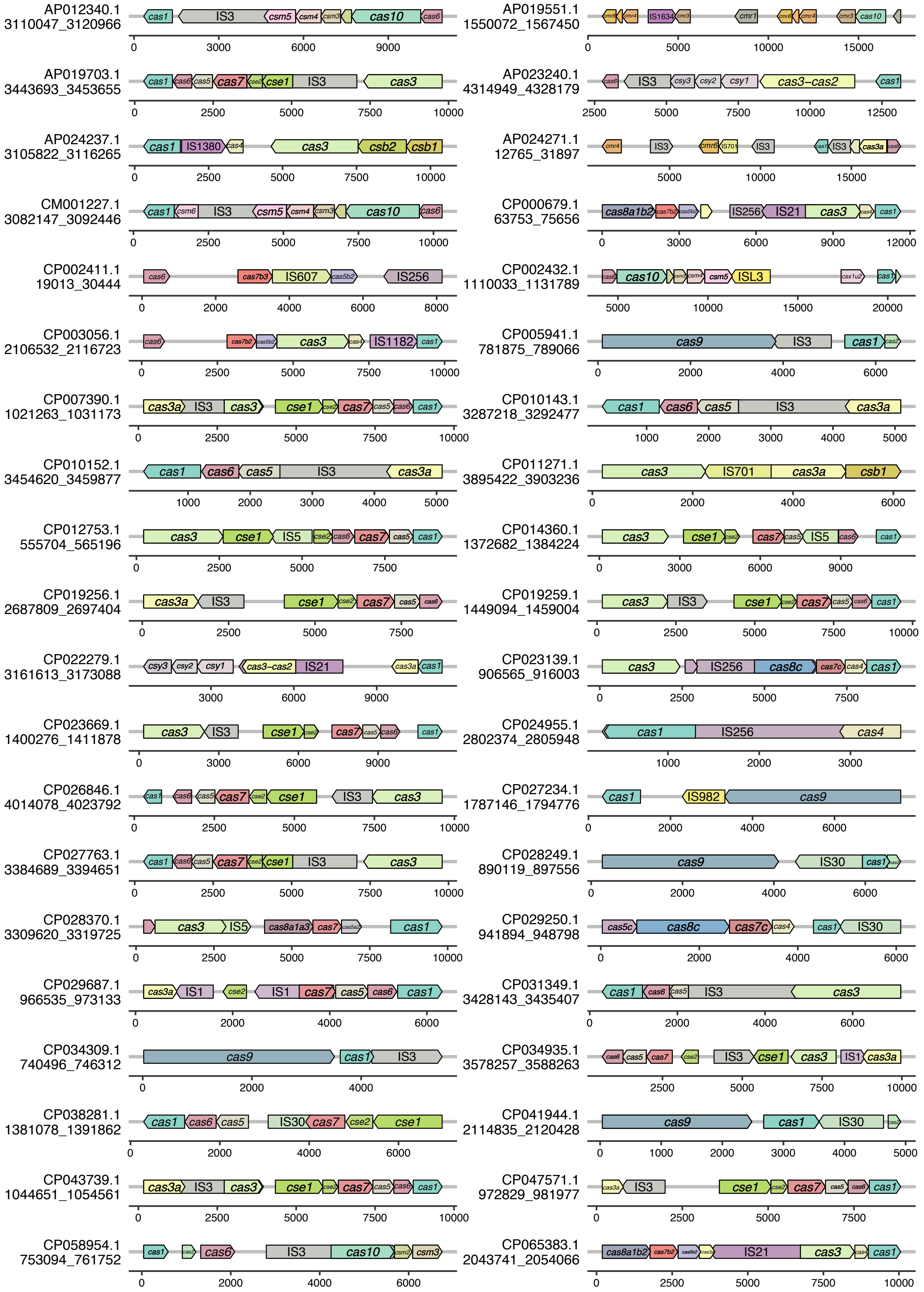
AP012340.1 3110047_3120966
cas1 IS3 csm5 csm4 csm3 cas10 cas6
AP019551.1 1550072_1567450
IS1634 cmr1 cmr6 cmr4 cmr3 cas10
AP019703.1 3443693_3453655
cas1 cas6 cas5 cas7 cse1 IS3 cas3
AP023240.1 4314949_4328179
cas6 IS3 csy3 csy2 csy1 cas3–cas2 cas1
AP024237.1 3105822_3116265
cas1 IS1380 cas4 cas3 csb2 csb1
AP024271.1 12765_31897
cmr4 IS3 cmr6 IS701 IS3 IS3 cas3a
CM001227.1 3082147_3092446
cas1 csm6 IS3 csm5 csm4 csm3 cas10 cas6
CP000679.1 63753_75656
cas8a1b2 cas7b2 cas5b2 IS256 IS21 cas3 cas1
CP002411.1 19013_30444
cas6 cas7b3 IS607 cas5b2 IS256
CP002432.1 1110033_1131789
cas10 csm5 ISL3
CP003056.1 2106532_2116723
cas6 cas7b2 cas5b2 cas3 IS1182 cas1
CP005941.1 781875_789066
cas9 IS3 cas1
CP007390.1 1021263_1031173
cas3a IS3 cas3 cse1 cas7 cas5 cas6 cas1
CP010143.1 3287218_3292477
cas1 cas6 cas5 IS3 cas3a
CP010152.1 3454620_3459877
cas1 cas6 cas5 IS3 cas3a
CP011271.1 3895422_3903236
cas3 IS701 cas3a csb1
CP012753.1 555704_565196
cas3 cse1 IS5 cse2 cas6 cas7 cas5 cas1
CP014360.1 1372682_1384224
cas3 cse1 cas7 cas5 IS5 cas6 cas1
CP019256.1 2687809_2697404
cas3a IS3 cse1 cse2 cas7 cas5 cas6
CP019259.1 1449094_1459004
cas3 IS3 cse1 cas7 cas5 cas6 cas1
CP022279.1 3161613_3173088
csy3 csy2 csy1 cas3–cas2 IS21 cas3a cas1
CP023139.1 906565_916003
cas3 IS256 cas8c cas7c cas4 cas1
CP023669.1 1400276_1411878
cas3 IS3 cse1 cas7 cas5 cas6 cas1
CP024955.1 2802374_2805948
cas1 IS256 cas4
CP026846.1 4014078_4023792
cas1 cas6 cas5 cas7 cse2 cse1 IS3 cas3
CP027234.1 1787146_1794776
cas1 IS982 cas9
CP027763.1 3384689_3394651
cas1 cas6 cas5 cas7 cse1 IS3 cas3
CP028249.1 890119_897556
cas9 IS30 cas1
CP028370.1 3309620_3319725
cas3 IS5 cas8a1a3 cas7 cas1
CP029250.1 941894_948798
cas5c cas8c cas7c cas4 cas1 IS30
CP029687.1 966535_973133
cas3a IS1 cse2 IS1 cas7 cas5 cas6 cas1
CP031349.1 3428143_3435407
cas1 cas6 cas5 IS3 cas3
CP034309.1 740496_746312
cas9 cas1 IS3
CP034935.1 3578257_3588263
cas6 cas5 cas7 cse2 IS3 cse1 cas3 IS1 cas3a
CP038281.1 1381078_1391862
cas1 cas6 cas5 IS30 cas7 cse2 cse1
CP041944.1 2114835_2120428
cas9 cas1 IS30
CP043739.1 1044651_1054561
cas3a IS3 cas3 cse1 cas7 cas5 cas6 cas1
CP047571.1 972829_981977
cas3a IS3 cse1 cse2 cas7 cas5 cas6 cas1
CP058954.1 753094_761752
cas1 cas6 IS3 cas10 csm2 csm3
CP065383.1 2043741_2054066
cas8a1b2 cas7b2 cas5b2 IS21 cas3 cas1

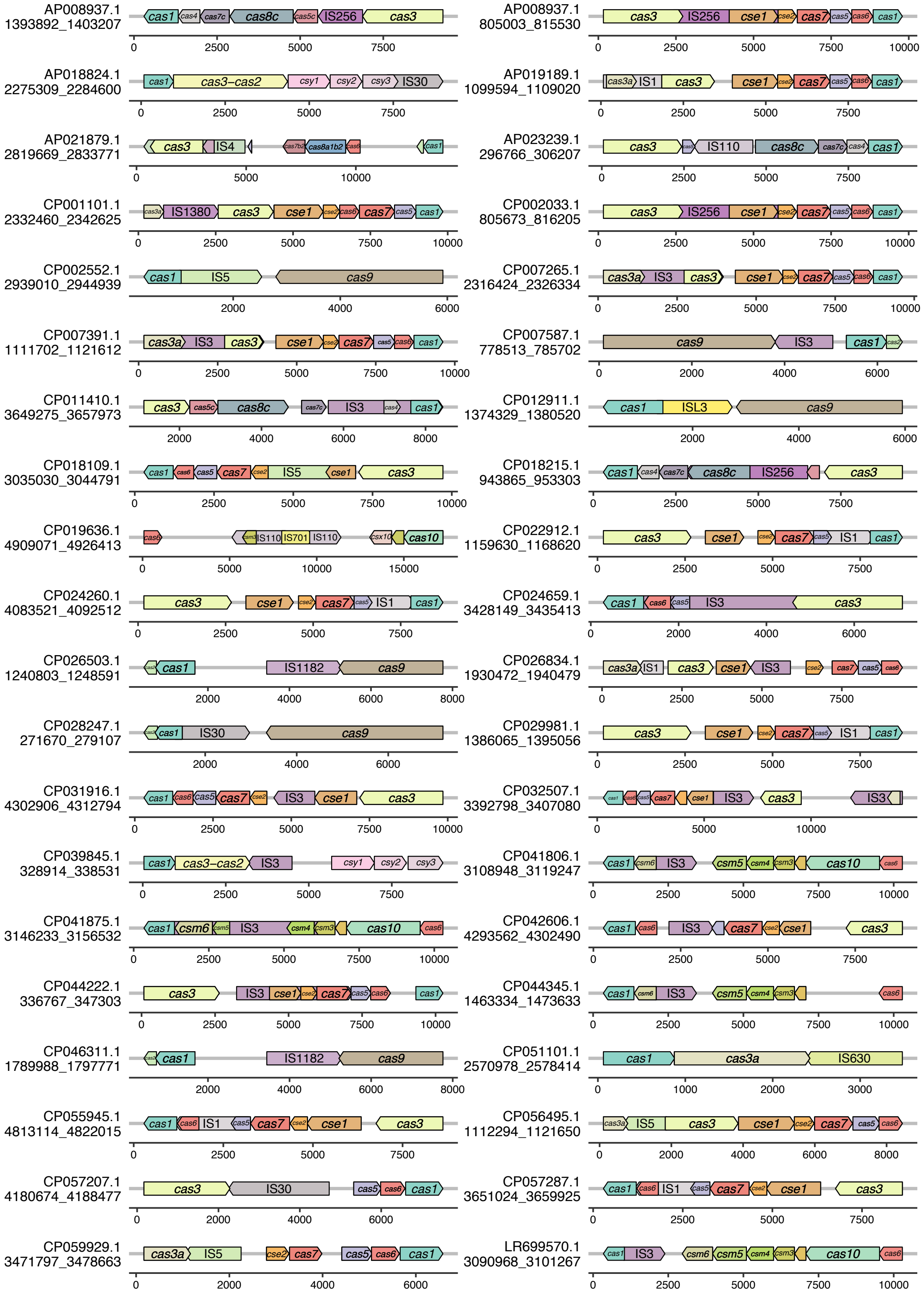
AP008937.1
1393892_1403207
cas1 cas4 cas7c cas8c cas5c IS256 cas3
AP008937.1
805003_815530
cas3 IS256 cse1 cse2 cas7 cas5 cas6 cas1
AP018824.1
2275309_2284600
cas1 cas3–cas2 csy1 csy2 csy3 IS30
AP019189.1
1099594_1109020
cas3a IS1 cas3 cse1 cse2 cas7 cas5 cas6 cas1
AP021879.1
2819669_2833771
cas3 IS4 cas7b cas8a1b2 cas6 cas1
AP023239.1
296766_306207
cas3 IS110 cas8c cas7c cas4 cas1
CP001101.1
2332460_2342625
cas3a IS1380 cas3 cse1 cse2 cas6 cas7 cas5 cas1
CP002033.1
805673_816205
cas3 IS256 cse1 cse2 cas7 cas5 cas6 cas1
CP002552.1
2939010_2944939
cas1 IS5 cas9
CP007265.1
2316424_2326334
cas3a IS3 cas3 cse1 cse2 cas7 cas5 cas6 cas1
CP007391.1
1111702_1121612
cas3a IS3 cas3 cse1 cse2 cas7 cas5 cas6 cas1
CP007587.1
778513_785702
cas9 IS3 cas1 cas2
CP011410.1
3649275_3657973
cas3 cas5c cas8c cas7c IS3 cas4 cas1
CP012911.1
1374329_1380520
cas1 ISL3 cas9
CP018109.1
3035030_3044791
cas1 cas6 cas5 cas7 cse2 IS5 cse1 cas3
CP018215.1
943865_953303
cas1 cas4 cas7c cas8c IS256 cas3
CP019636.1
4909071_4926413
cas6 IS110 IS701 IS110 csx10 cas10
CP022912.1
1159630_1168620
cas3 cse1 cse2 cas7 cas5 IS1 cas1
CP024260.1
4083521_4092512
cas3 cse1 cse2 cas7 cas5 IS1 cas1
CP024659.1
3428149_3435413
cas1 cas6 cas5 IS3 cas3
CP026503.1
1240803_1248591
cas1 IS1182 cas9
CP026834.1
1930472_1940479
cas3a IS1 cas3 cse1 IS3 cse2 cas7 cas5 cas6
CP028247.1
271670_279107
cas1 IS30 cas9
CP029981.1
1386065_1395056
cas3 cse1 cse2 cas7 cas5 IS1 cas1
CP031916.1
4302906_4312794
cas1 cas6 cas5 cas7 cse2 IS3 cse1 cas3
CP032507.1
3392798_3407080
cas1 cas7 cse1 IS3 cas3 IS3
CP039845.1
328914_338531
cas1 cas3–cas2 IS3 csy1 csy2 csy3
CP041806.1
3108948_3119247
cas1 csm6 IS3 csm5 csm4 csm3 cas10 cas6
CP041875.1
3146233_3156532
cas1 csm6 csm5 IS3 csm4 csm3 cas10 cas6
CP042606.1
4293562_4302490
cas1 cas6 IS3 cas7 cse2 cse1 cas3
CP044222.1
336767_347303
cas3 IS3 cse1 cse2 cas7 cas5 cas6 cas1
CP044345.1
1463334_1473633
cas1 csm6 IS3 csm5 csm4 csm3 cas6
CP046311.1
1789988_1797771
cas1 IS1182 cas9
CP051101.1
2570978_2578414
cas1 cas3a IS630
CP055945.1
4813114_4822015
cas1 cas6 IS1 cas5 cas7 cse2 cse1 cas3
CP056495.1
1112294_1121650
cas3a IS5 cas3 cse1 cse2 cas7 cas5 cas6
CP057207.1
4180674_4188477
cas3 IS30 cas5 cas6 cas1
CP057287.1
3651024_3659925
cas1 cas6 IS1 cas5 cas7 cse2 cse1 cas3
CP059929.1
3471797_3478663
cas3a IS5 cse2 cas7 cas5 cas6 cas1
LR699570.1
3090968_3101267
cas1 IS3 csm6 csm5 csm4 csm3 cas10 cas6

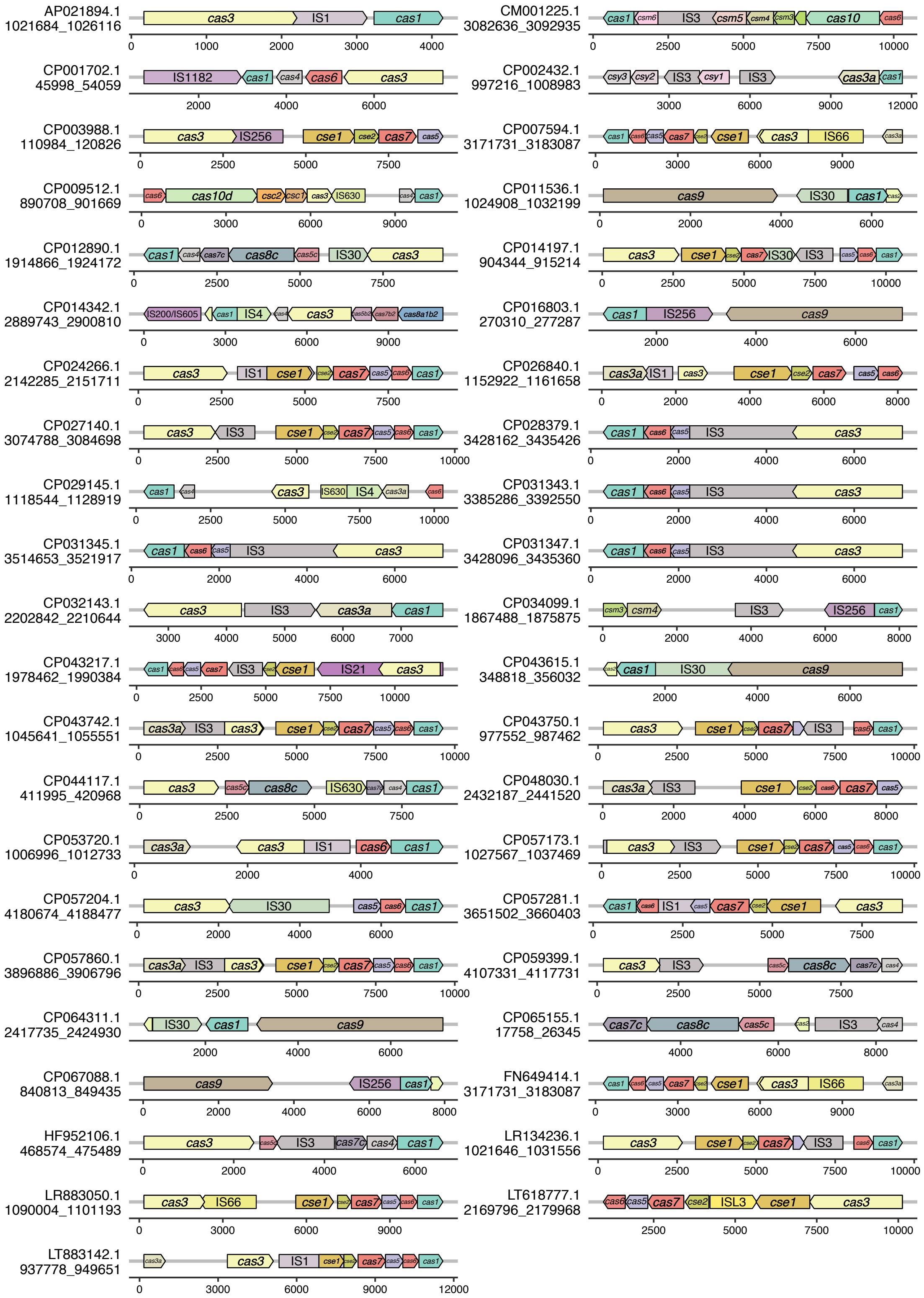

Figure S2. Identification of naturally occurring transpositions of ISs into *cas* genes using a highly sensitive software pipeline, ISEScan. A total of 163 transposition events are shown above. Each IS element in the figures represents a unique IS family.

Horizontal axes indicate the relative position of each gene or IS within the targeted genomic region.

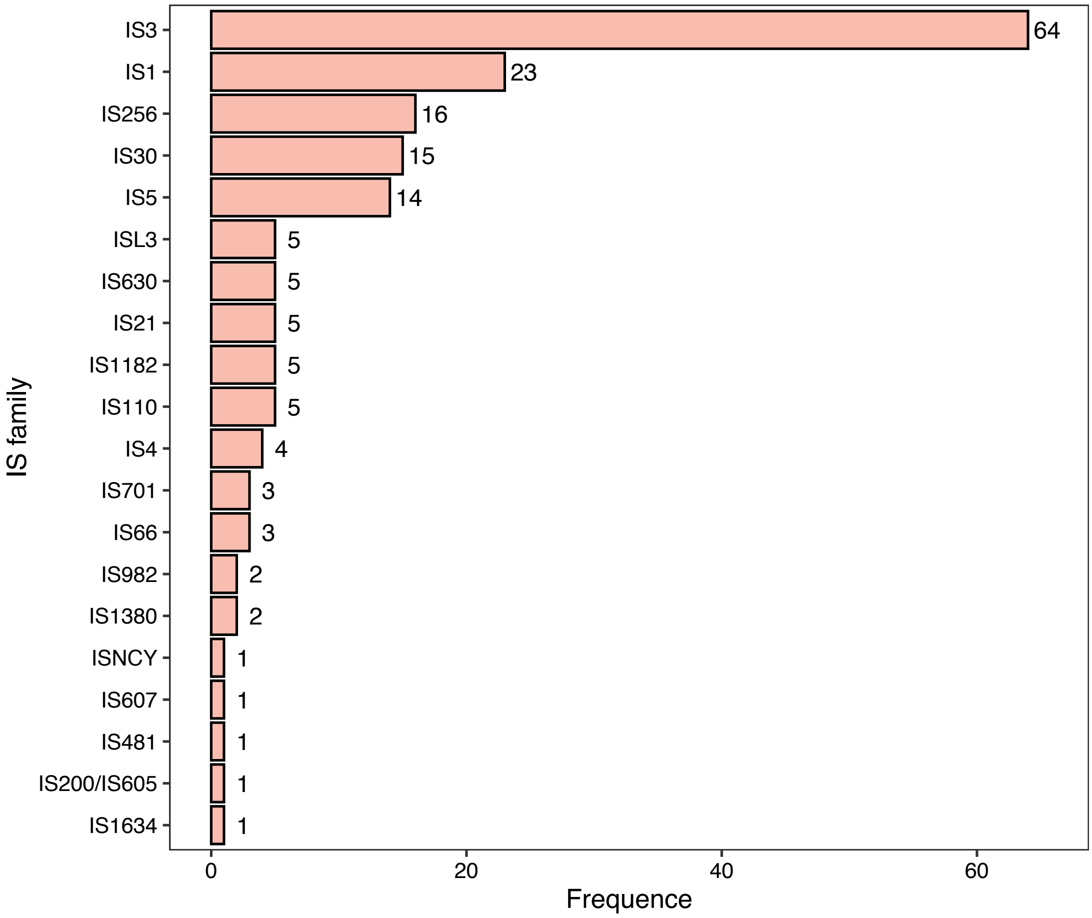

Figure S3. Analysis of ISs engagement in 163 cases of naturally occurring transpositions of ISs into *cas* genes. It is important to note that certain transposition events within the analyzed cases involve the participation of multiple IS families, rather than a single IS family.

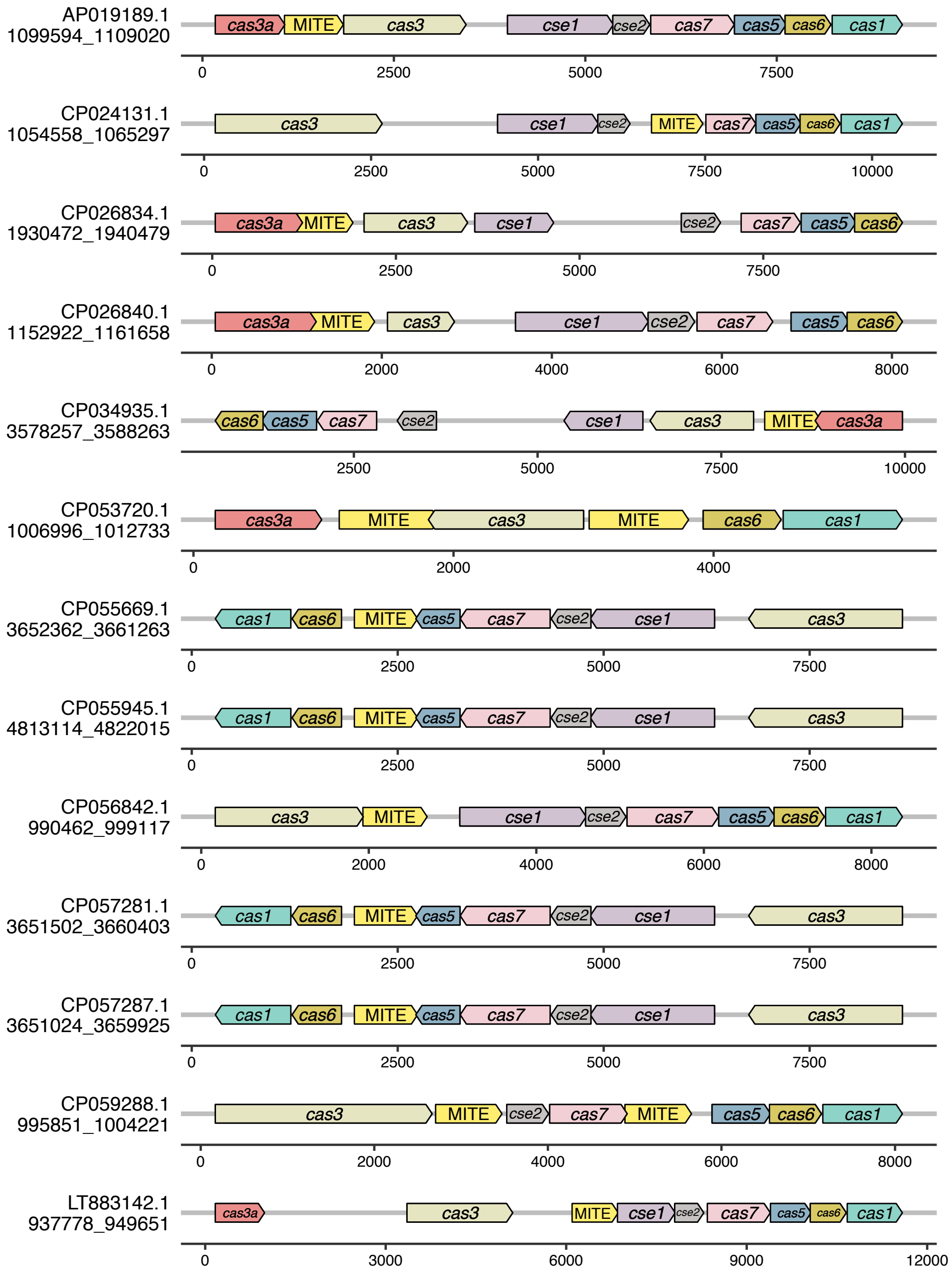

Figure S4. Naturally occurring transpositions of MITEs into *cas* genes. Horizontal axes indicate the relative position of each gene or MITE within the targeted genomic region.

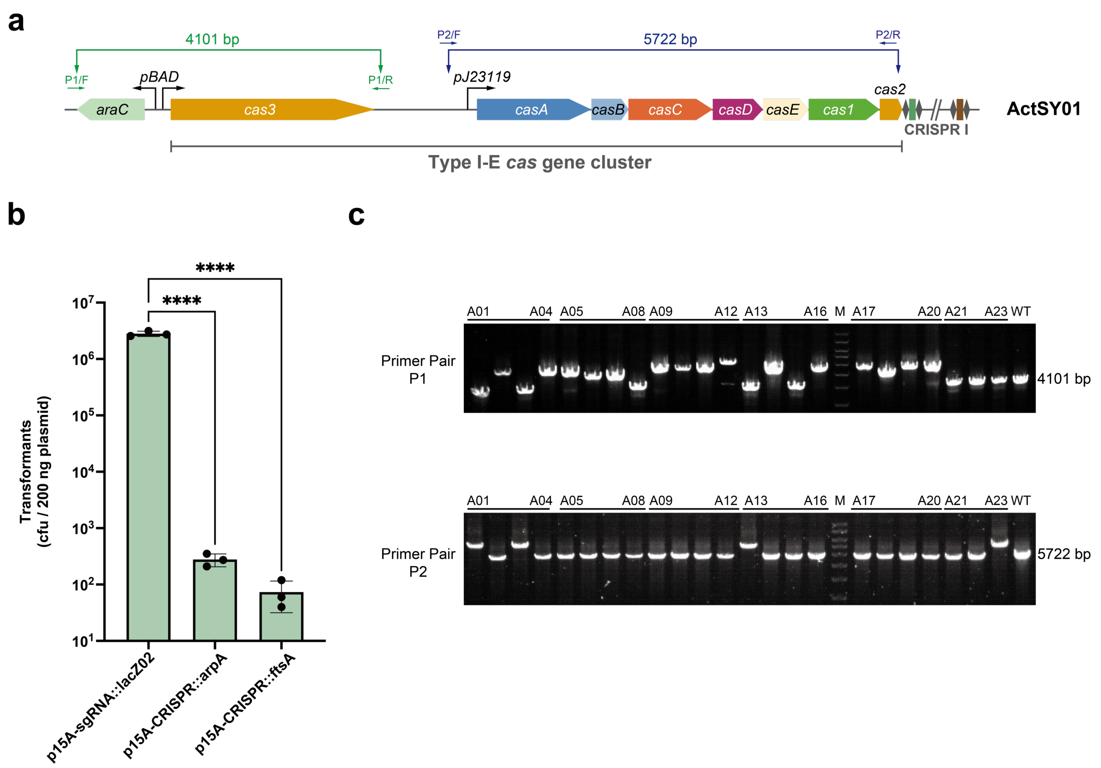

Figure S5. ISs mediate the fitness trade-off between the benefits of acquiring plasmids and the genotoxicity induced by the type I-E CRISPR/Cas system of *E. coli* DH10B under antibiotic pressure. a Schematic of the engineered type I-E CRISPR-Cas locus of *E. coli* DH10B. The strain ActSY01 was generated by replacing the native promoters of *casABCDE12* operon and *cas3* with a constitutive promoter J23119 and an arabinose-inducible promoter (pBAD), respectively. Primer pairs P1 and P2 were designed to amplify the entire *cas* cluster of the strain ActSY01; expected amplicon sizes are shown. **b** In the presence of L-arabinose induction, transformation efficiencies of p15A-sgRNA::lacZ02 (control, lacking the targeting spacer for the type I-E CRISPR/Cas system), p15A-CRISPR::arpA (targeting the non-essential gene *arpA* of the strain ActSY01) and p15A-CRISPR::ftsA (targeting the essential gene *ftsA* of the strain ActSY01) into the strain ActSY01 were determined through counting CFUs per 200 ng of plasmids. Error bars denote means ± S.D. from three biological replicates;

****, $P < 0.0001$, using two-tailed unpaired t-test; P = 9.03e-05, 9.03e-05 (from top to bottom). Source data are provided as a Source Data file. **c** Colony PCR screening with primer pairs P1 and P2 for IS insertions into the *cas* cluster of the 23 escapers (A01-A23). Lanes "WT", control amplicons with the strain ActSY01 template; larger PCR products suggest IS transposition events. Except for samples A8, A15, A21 and A22, IS insertions were detected within the *cas* genes of all other 19 samples. Detailed information regarding the IS types involved in transposition and their resulting TSD sequences can be found in Table S4. The experiment was conducted independently three times and yielded consistent results. Source data are provided as a Source Data file.

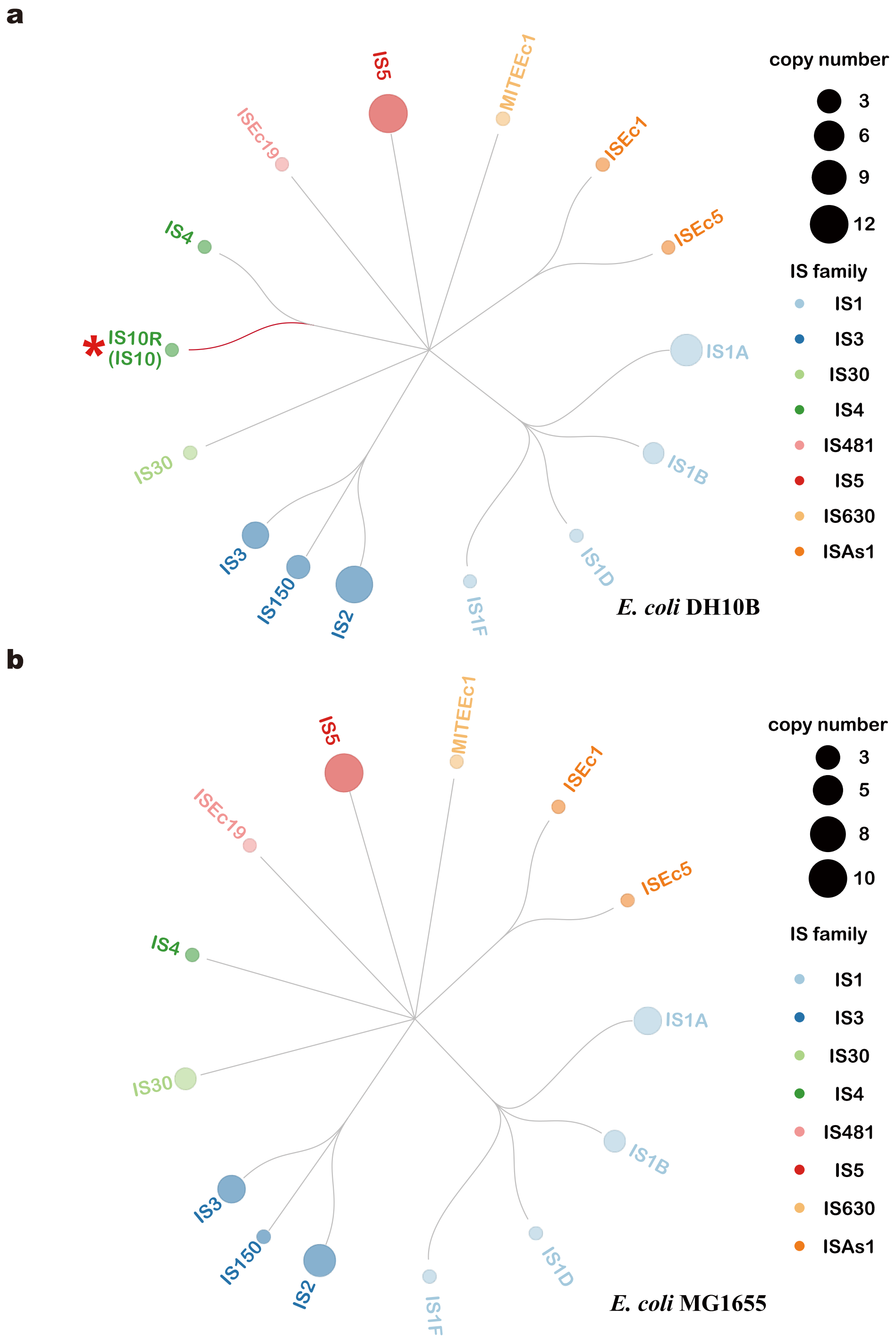

Figure S6. Analysis of the diversity and copy number of different insertion sequences within *E. coli* DH10B (a) and *E. coli* MG1655 (b). Each colored circle represents a specific IS family, and the circle size reflects the copy number of the IS element on the chromosome with a larger size indicating greater copy number. Except for the IS10 element, the same ISs were detected in both strains.

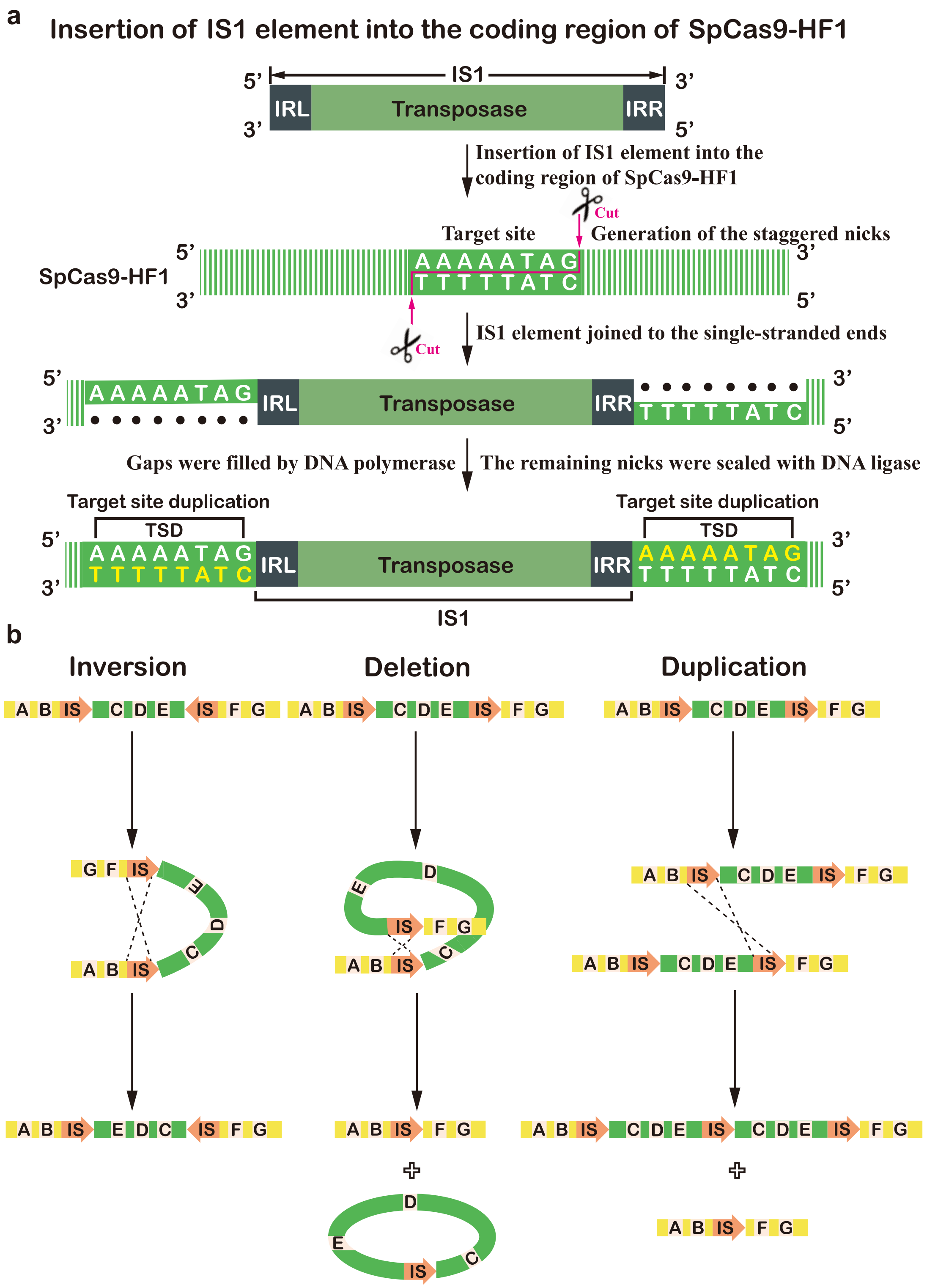

Figure S7. Schematic illustrations of the process for ISs insertion into the coding region of the Cas protein (a) and of the DNA rearrangements resulting from homologous recombination between two identical IS elements (b). a Transposition

example showing the insertion of IS1 into the coding region of SpCas9-HF1. IS1 is composed of the internal transposase flanked at both ends by two inverted repeats (IRL and IRR). The transposase cleaves at the target site within the coding region of SpCas9-HF1 and introduces the staggered nicks, followed by joining to the resulting single-stranded ends. Subsequently, the DNA gaps are filled by DNA polymerase, and the remaining nicks are sealed with DNA ligase, generating an 8 bp or 9 bp target site duplication around IS1 within the SpCas9-HF1 coding region. **b** Inversion, deletion, and duplication of the DNA segment resulting from recombination between two copies of the same IS element. The intervening sequence is likely inverted by homologous recombination when an IS element is inserted into the target sequence in two opposite orientations, whereas copies of the IS element in the same orientation may trigger either deletion or duplication of the intervening sequence.

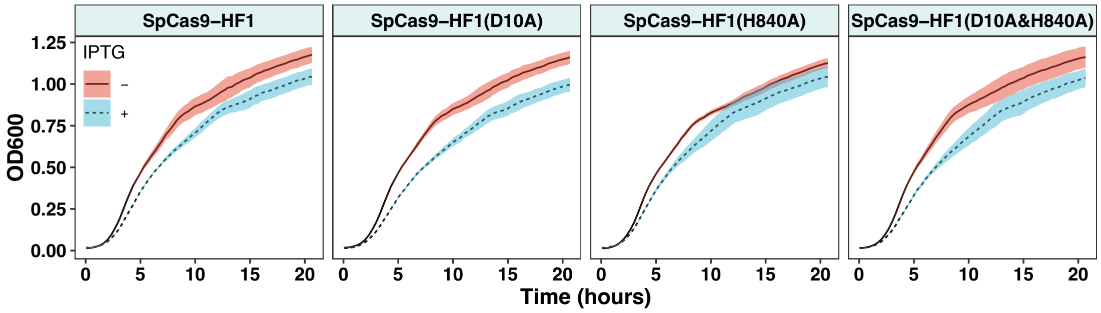

Figure S8. The mere induction of Cas protein expression does not lead to noticeable growth defects in the host strains. Growth curves of the strains encoding different Cas protein variants under both IPTG-induced and non-induced conditions. Shaded areas indicate the means ± S.D. from five biological replicates. Source data are provided as a Source Data file.

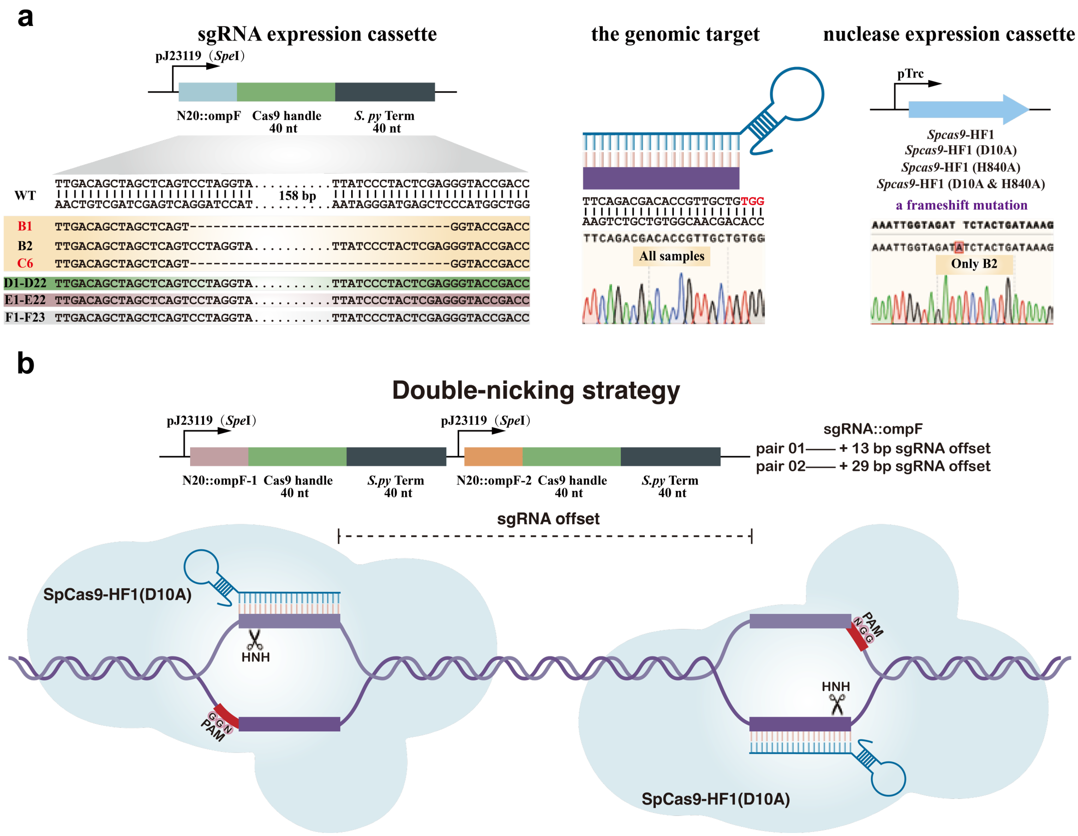

Figure S9. Identification of mutations in the CRISPR-tolerant mutants (a) and schematic illustration of the CRISPR double-nicking strategy (b). a Except for the colonies with IS insertions into the *cas* gene, all the remaining CRISPR-tolerant mutants (targeted by sgRNA::ompF) described in Figure 4c were further analyzed to determine the other factors that enabled escape from CRISPR-Cas interference. All genetic components necessary for the proper functioning of CRISPR-Cas system, including the sgRNA expression cassette, the genomic target, and the nuclease expression cassette, were sequenced by the Sanger method. Sequence analysis revealed a deletion mutation within the sgRNA expression cassettes of the mutant B1 and C6, as well as a frameshift mutation within the coding region of SpCas9-HF1 of the mutant B2, all of which led to their escape from SpCas9-HF1-mediated DSB. However, in contrast to SpCas9-HF1, the variants containing mutated nuclease domains, including

SpCas9-HF1 (D10A), SpCas9-HF1 (H840A), and SpCas9-HF1 (D10A & H840A), were unable to induce any detectable mutations which might potentially compromise CRISPR-Cas systems, indicating that they were incapable of triggering genotoxic stress. **b** The CRISPR double-nicking strategy with two pair of offset sgRNAs. The schematic depiction of the staggered DSB formation by SpCas9-HF1 (D10A) nickase in the presence of paired sgRNAs.

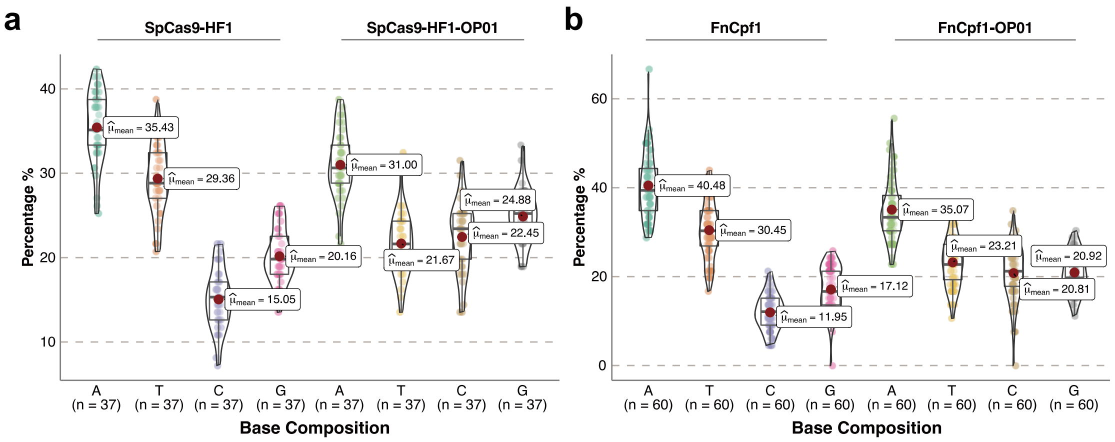

Figure S10. Comparison of base composition between the coding regions of SpCas9-HF1 and SpCas9-HF1-OP01 (a), and FnCpf1 and FnCpf1-OP01 (b). To determine the local base composition features within a nucleotide sequence, the coding regions of SpCas9-HF1, SpCas9-HF1-OP01, FnCpf1, and FnCpf1-OP01 were individually divided into 37, 37, 60, and 60 fragments, respectively, and the strand bias in nucleotide composition was computed for each segment. The total number of samples analyzed was indicated under each boxplot (n = 37 for SpCas9-HF1 and SpCas9-HF1-OP01; n = 60 for FnCpf1 and FnCpf1-OP01). The boxplots show medians (midlines), interquartile ranges (boxes) and ranges (whiskers).

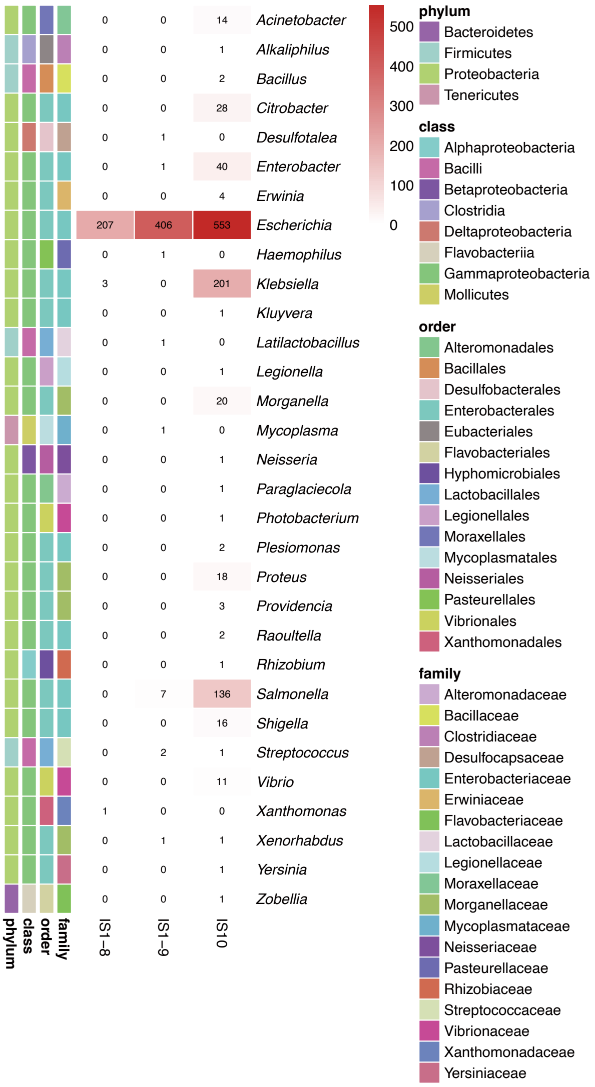

Figure S11. Enrichment analysis of the naturally occurring transposition events of IS1 and IS10 across bacterial taxonomic groups. The number of genomes per genus in which IS1 or IS10 had been transposed is indicated by a color gradient, and the actual numbers are displayed in boxes.

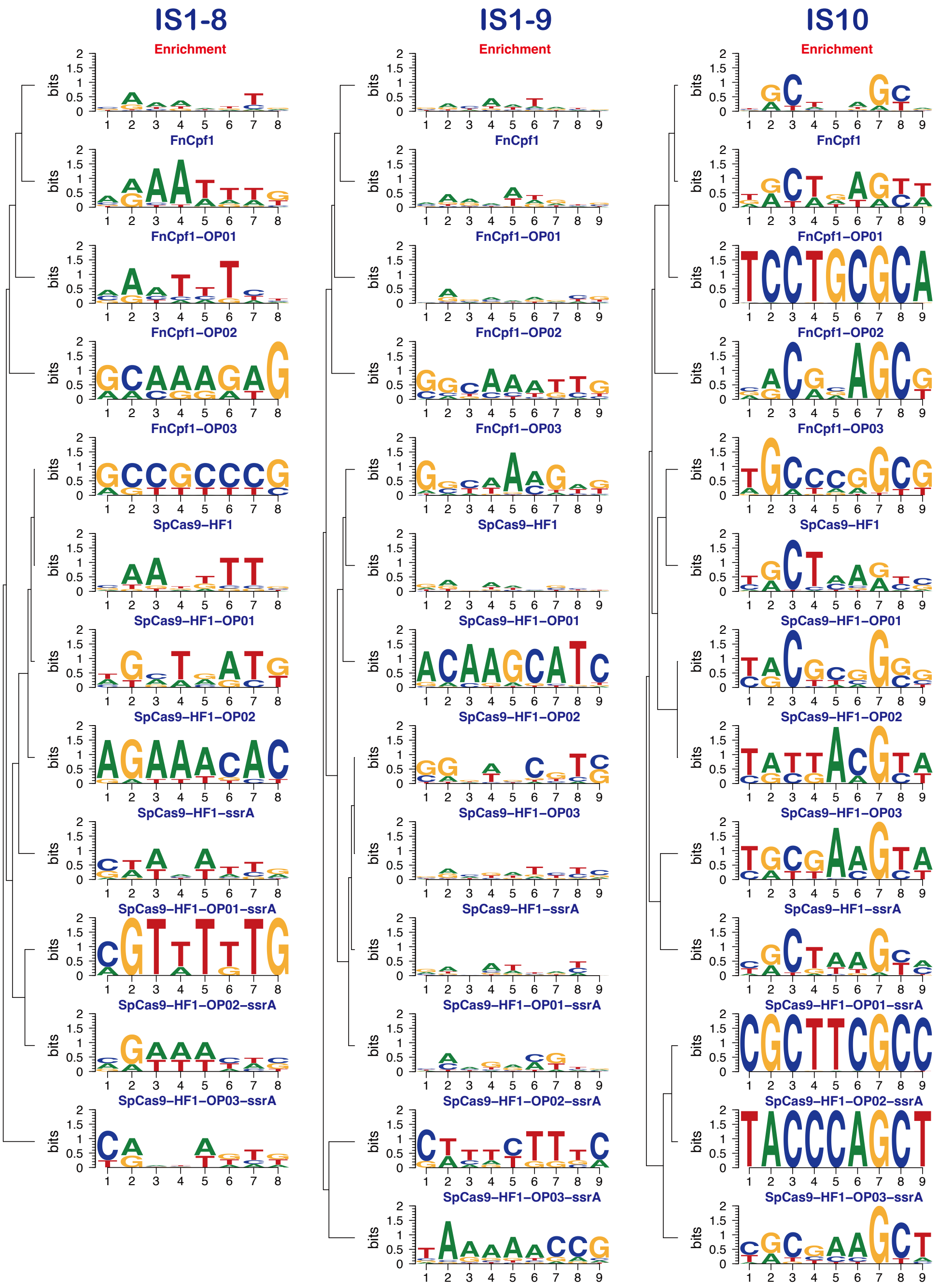

Figure S12. Comprehensive comparison of various TSD motifs generated by IS1 and IS10 within all constructed *cas* genes. Naturally occurring TSD motifs are labeled with “Enrichment” in red text (in reference to their enrichment/retrieval through bioinformatics analysis), and the name of each *cas* gene from which TSD sequences were collected is marked above the corresponding sequence logo.

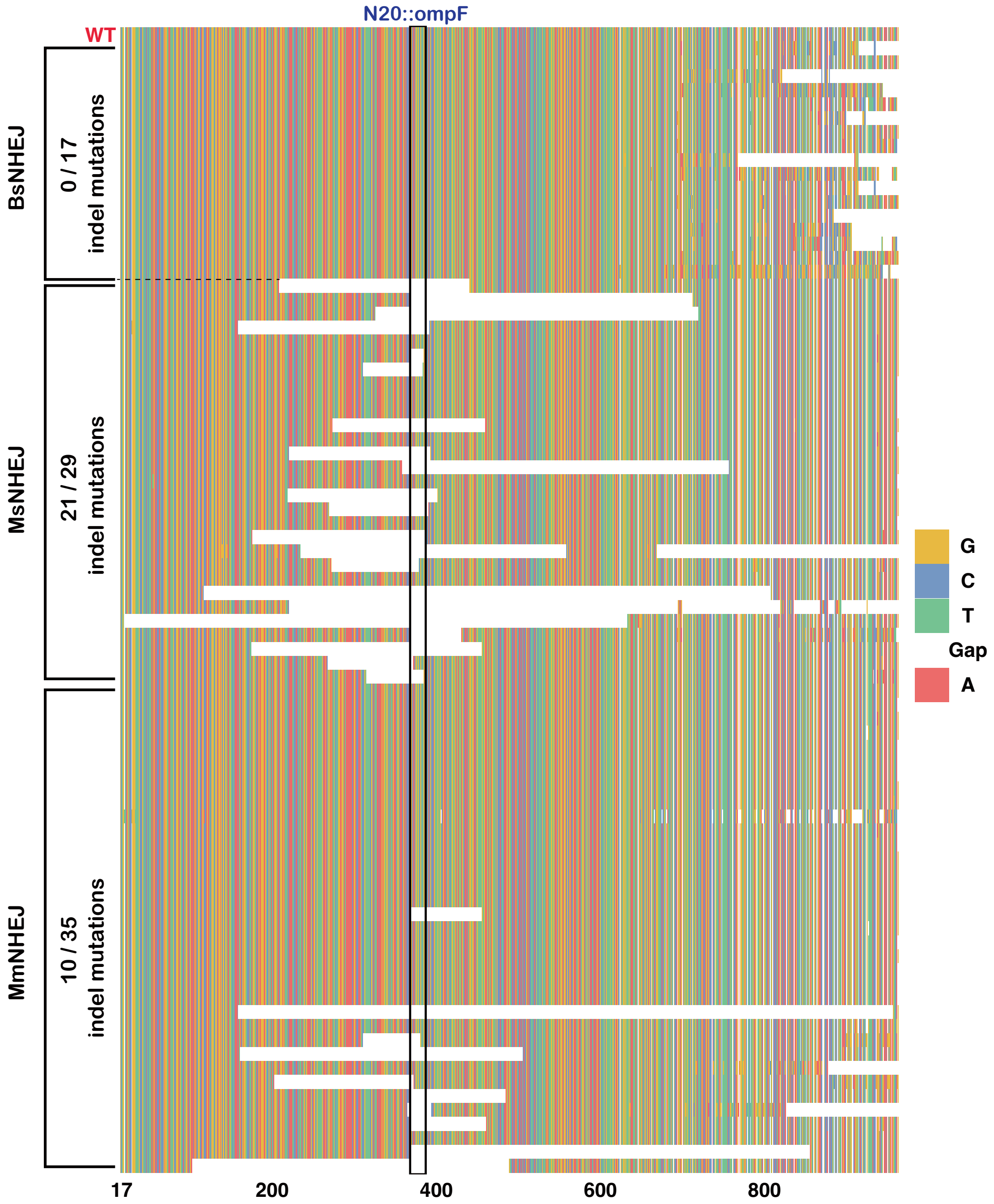

Figure S13. Detection of targeted indel mutations induced by NHEJ repair. The sgRNA targeting site (N20::ompF) is marked with a black rectangle, and the sequencing result from WT (without CRISPR/Cas-induced DSB) served as the control. A series of pixels was used to represent each nucleotide sequence, and the four distinct colors represent each base (A, T, C, G). Note that only the confidence intervals of sequencing results should be considered.

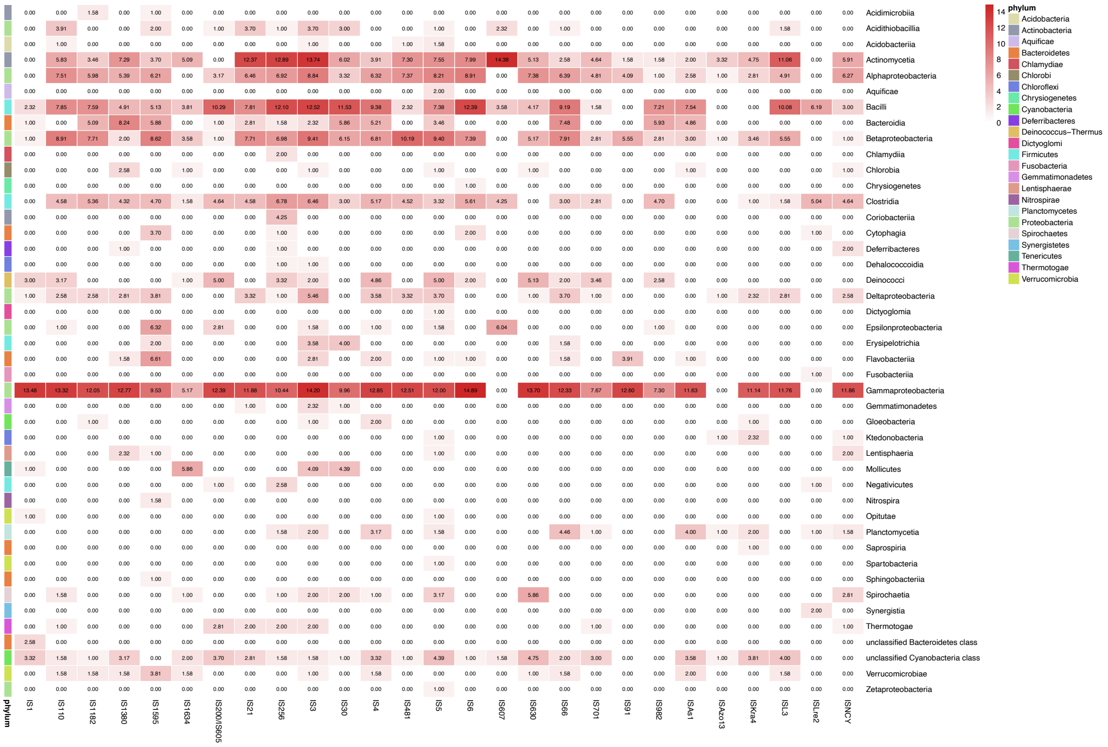

Figure S14. Diversity distribution of different IS families across bacterial taxonomic groups. The number of genomes per class containing a specific IS family is indicated by the color gradient from white (less) to firebrick red (more). Due to the different scales of the values within the heatmap analysis, the numbers in boxes were log-transformed (log2(x+1)) for better visualization.

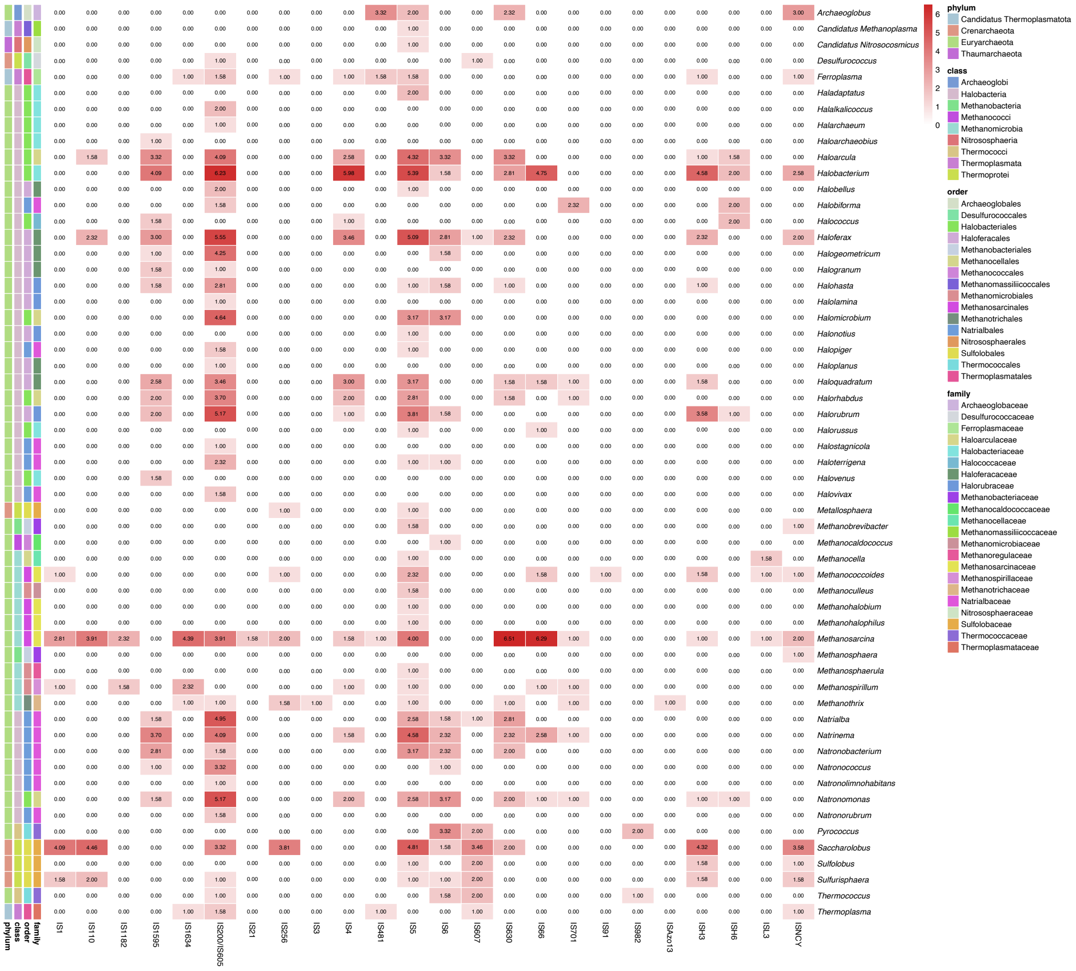

Figure S15. Diversity distribution of different IS families across archaeal taxonomic groups. The number of genomes per genus containing a specific IS family is indicated by the scaled color coding. The values in boxes were log-transformed (log2(x+1)) for better visualization.

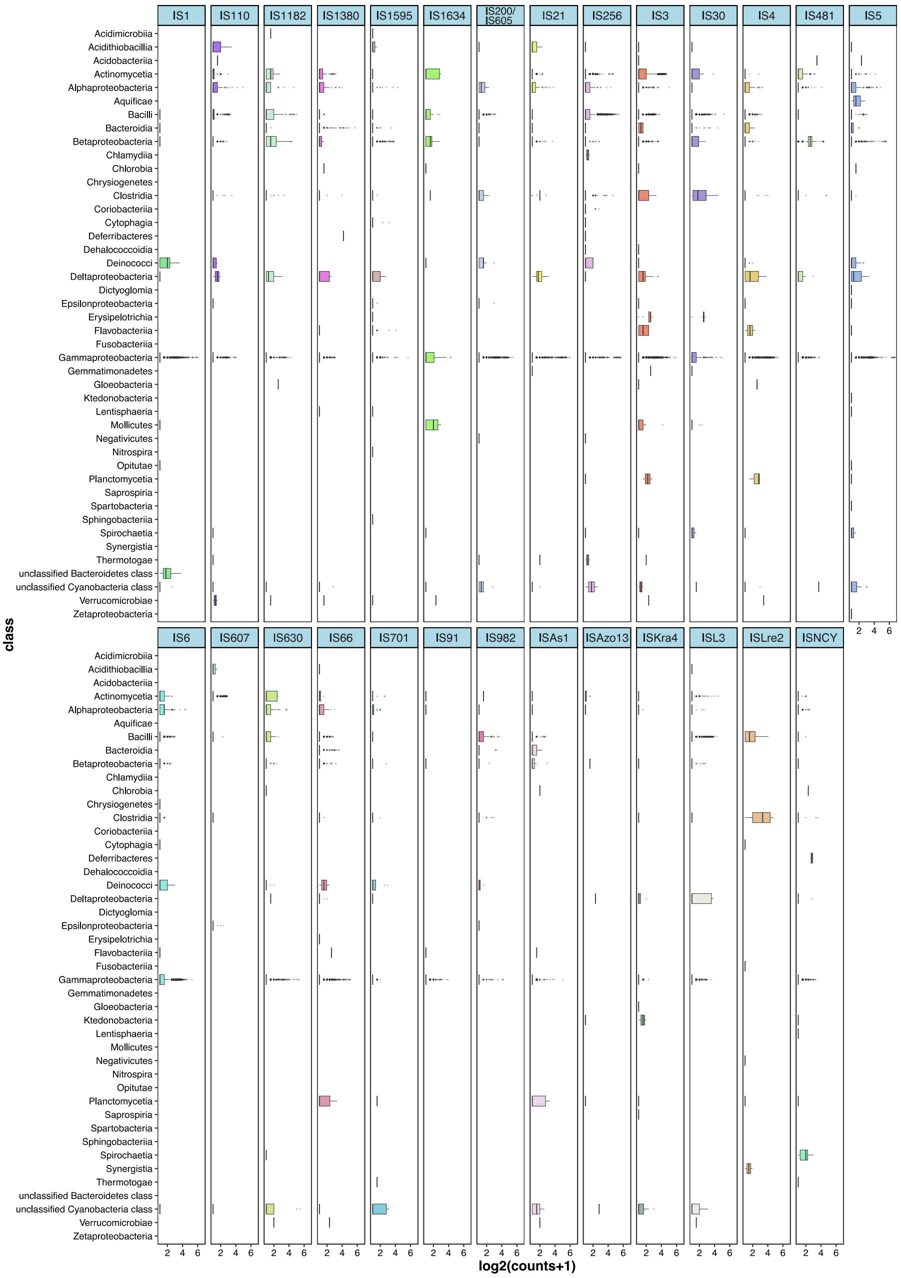

Figure S16. Distribution of different IS family counts among bacterial taxonomic groups. The boxplot diagram shows the counts per genome of each IS family across various bacterial genomes at the class level. The counts were log-transformed (log2(x+1)) for enhanced resolution, and only non-zero counts were plotted. The boxplots show medians (midlines), interquartile ranges (boxes) and ranges (whiskers). As the figure includes multiple boxplots, the number of genomes analyzed in each individual boxplot is provided in Supplementary Data 3.

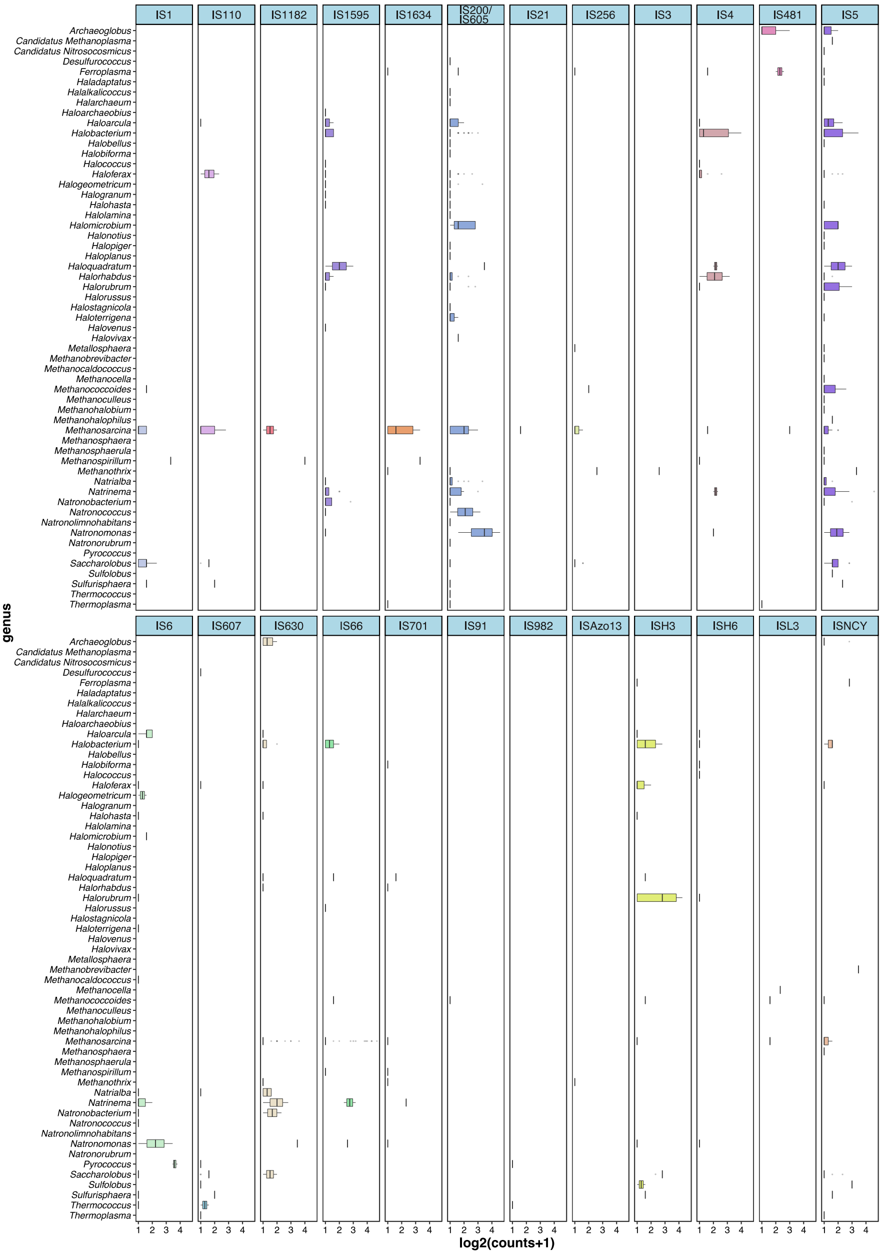

Figure S17. Distribution of different IS family counts among archaeal taxonomic groups. The boxplot diagram shows the counts per genome of each IS family across various archaeal genomes at the genus level. The count distributions were plotted on a log2(x+1) scale (x-axis) for better data visualization, and only non-zero values were plotted. The boxplots show medians (midlines), interquartile ranges (boxes) and ranges (whiskers). As the figure includes multiple boxplots, the number of genomes analyzed in each individual boxplot is provided in Supplementary Data 4.

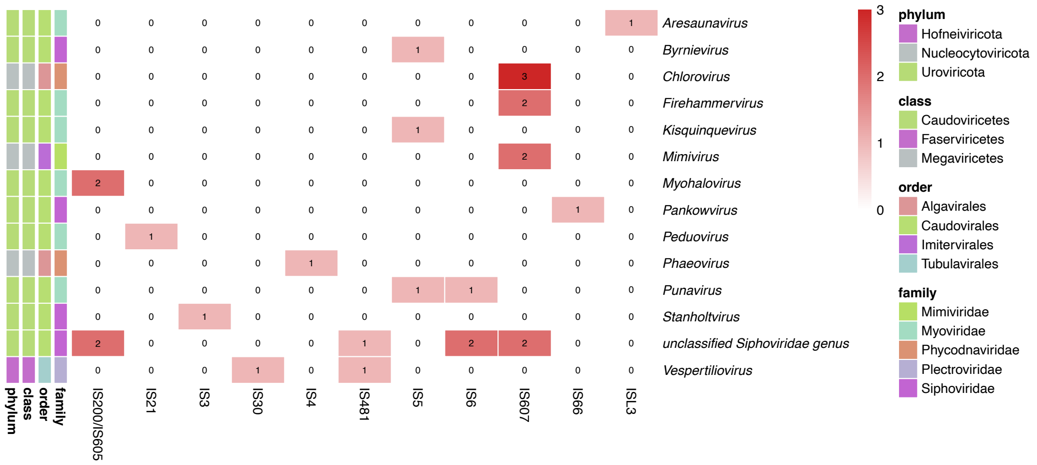

Figure S18. Diversity distribution of different IS families across viral taxonomic groups. The number of genomes per genus containing a specific IS family is indicated by a color gradient, and the numbers in boxes represent the actual values.

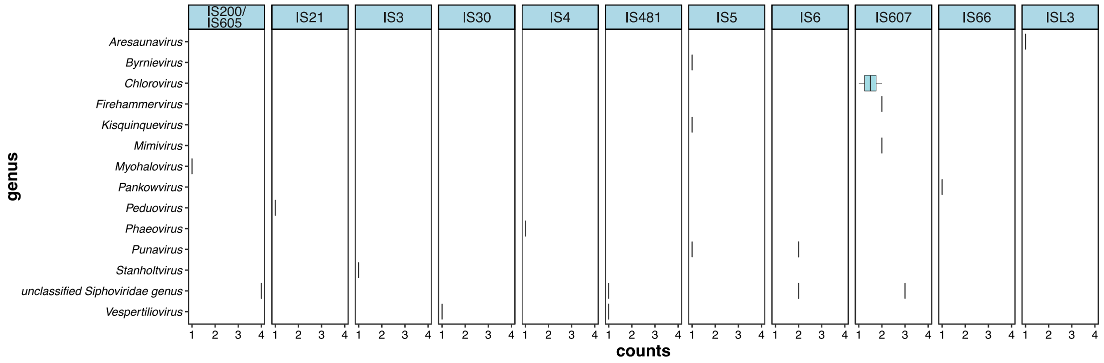

Figure S19. Distribution of different IS family counts among viral taxonomic groups. The boxplot diagram shows the counts per genome of each IS family across the various viral genomes at the genus level, and the horizontal coordinates represent the actual numbers. Only non-zero observations are shown. The boxplots show medians (midlines), interquartile ranges (boxes) and ranges (whiskers). As the figure includes multiple boxplots, the number of genomes analyzed in each individual boxplot is provided in Supplementary Data 5.

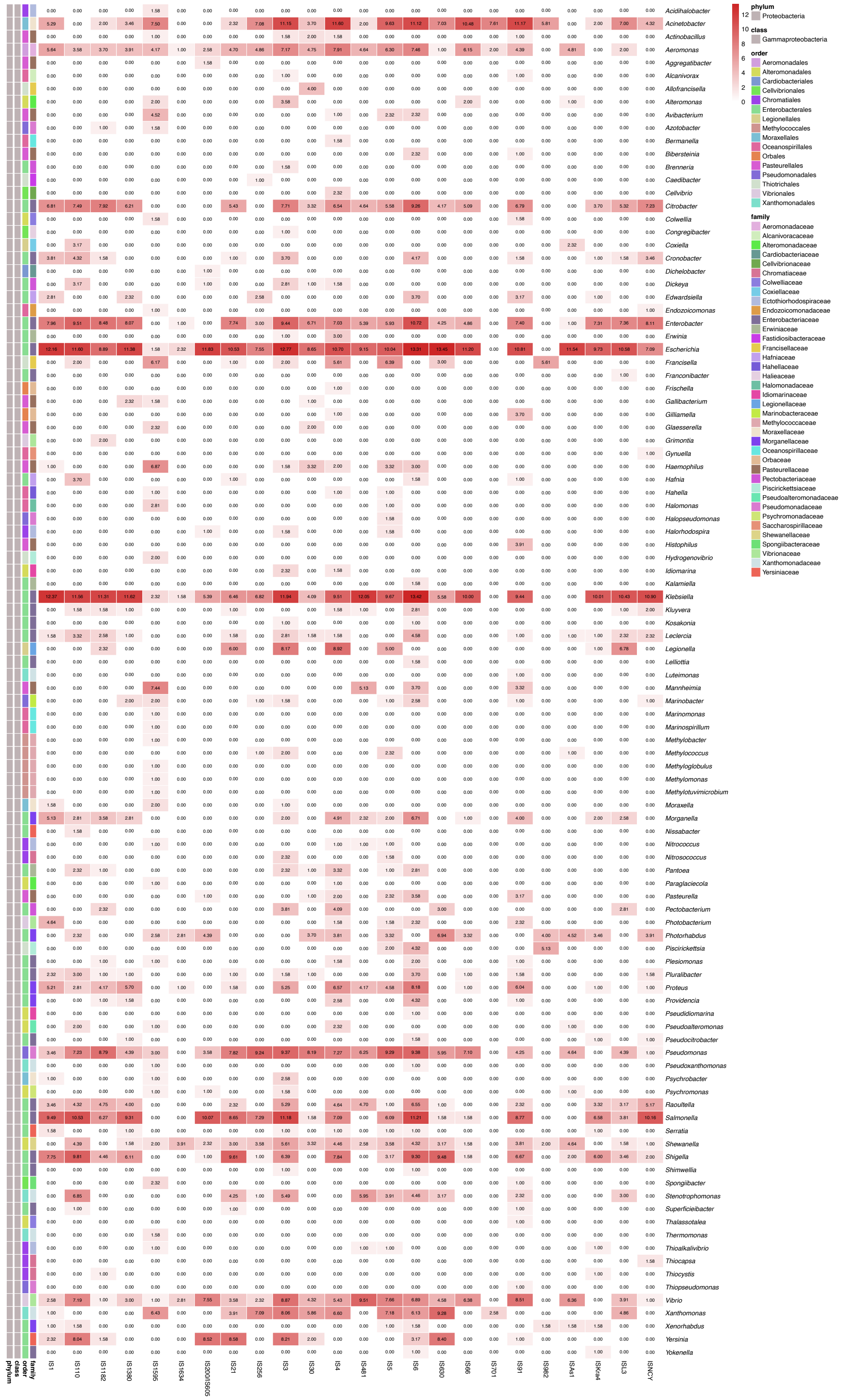

Figure S20. Diversity distribution of different IS families within the class Gammaproteobacteria. Heatmap analysis of the diversity distribution of different IS

families within the class Gammaproteobacteria at the genus level. The color gradient indicates the number of genomes per genus that contained the given IS family. The values in boxes were log-transformed (log2(x+1)) for greater clarity.

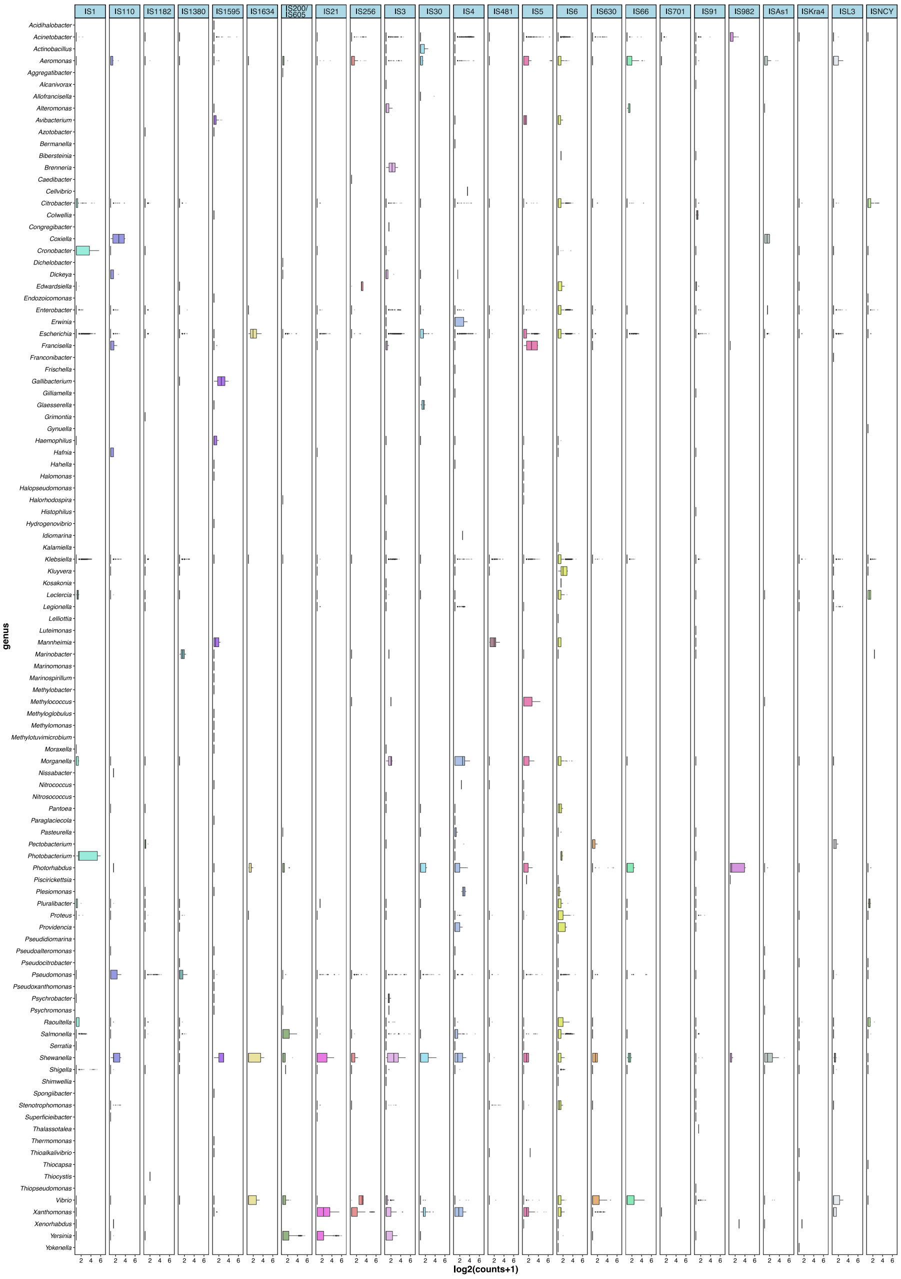

Figure S21. Distribution of different IS family counts among the class Gammaproteobacteria. Boxplot diagram of counts per genome for each IS family within Gammaproteobacteria genomes is shown at the genus level. On the x-axis, the counts were log-transformed (log2(x+1)), and only non-zero values were plotted. The boxplots show medians (midlines), interquartile ranges (boxes) and ranges (whiskers). As the figure includes multiple boxplots, the number of genomes analyzed in each individual boxplot is provided in Supplementary Data 6.

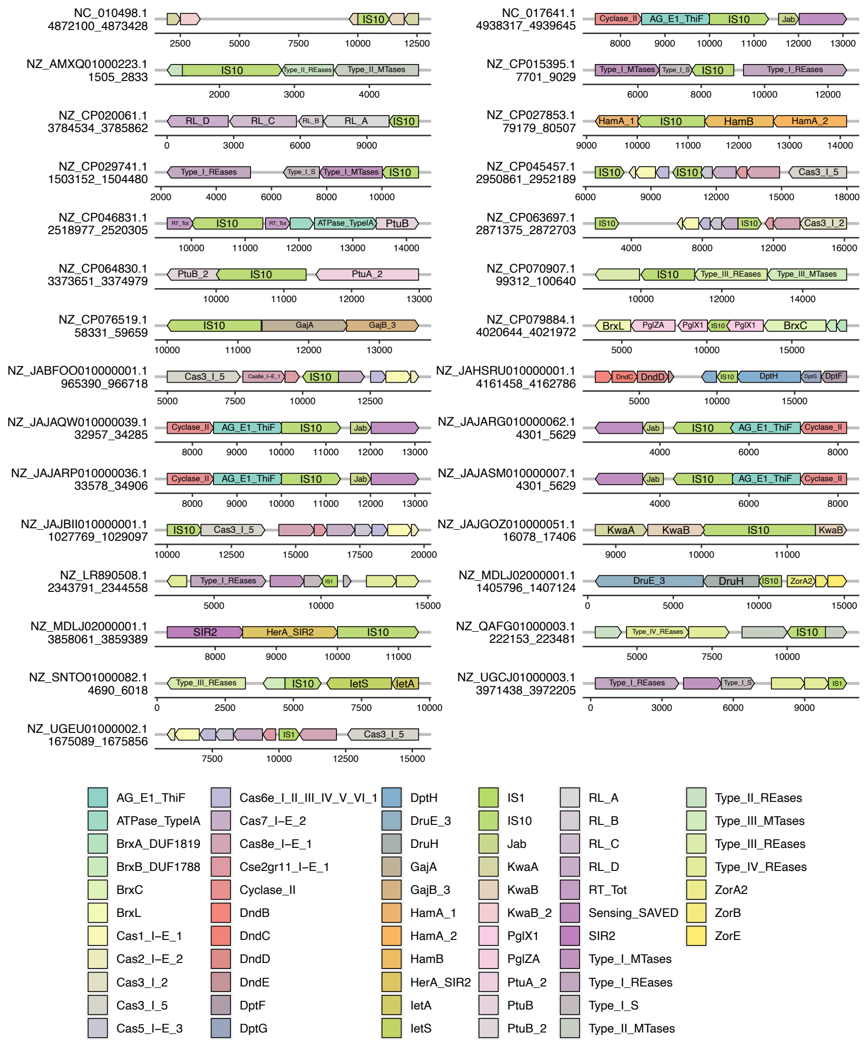

Figure S22. Schematic diagram of compromised defense systems resulting from naturally occurring transpositions of IS1 and IS10. All identified transposition events are shown.

Supplementary Tables

Table S1. Primers used in this study.

**Assembly cloning of the type I-E CRISPR-Cas system of *Erwinia amylovora* 7-3**	
EraCas-locus234-2-F	AGATCTCAGGCAGACTGCCG
EraCas-locus234-2-R	CTGCGCGTCAGACGGCATCC
EraCas-locus234-3-F	CTGCCTGTTACAAACGCTGT
EraCas-locus234-3-R	AGGTGGGCGGCAATAGTGAG
EraCas-locus234-4-F	GTGCAAGCCGTGAGCAGGCG
EraCas-locus234-4-R	AAGCTTGCCCAGTATTCTTC
T-Apr-IS10-Era-1-F	ATTATCAGCAGCACCTTGGGGAATCAAGCATGCGCACCACTGA GAGATCCCCTCATAAT
T-Apr-IS10-Era-1-R	TTATCATCCCCTTTTGCTGATCATGACCAATTTCTAGCCG
T-Apr-IS10-Era-2-F	CGGCTAGAAATTGGTCATGATCAGCAAAAGGGGATGATAA
T-Apr-IS10-Era-2-R	GAGCAATGTTCAAAGAAAGCTCATGAGCTCAGCCAATCGA
T-Apr-IS10-Era-3-F	TCGATTGGCTGAGCTCATGAGCTTTCTTTGAACATTGCTC
T-Apr-IS10-Era-3-R	GACGGGCATGAACCACATTACGCAGATCCGCCAGATGGATGG TGCGCACTGATGAATCCCCTAATG
T-Apr-pBAD-1-F	CCGTCTGATAATGGAATATAACGGTCGTAAAGGGAGCTTCTCG TTCTCCGCTCATGAGC
T-Apr-pBAD-1-R	CGCTCATGTCTAGACGATCGTCAGCAAAAGGGGATGATAA
T-Apr-pBAD-2-F	TTATCATCCCCTTTTGCTGACGATCGTCTAGACATGAGCG
T-Apr-pBAD-2-R	CTTACCCCAATAGCGGTAATACTCAGGCTGCGAATTCATTTTTT ATAACCTCCTTAGAG
T-EraP-pro-Ichl-F	AGGGCCGCAGATGCGACCCTTGTGTATCAAACAAGACGAACG CACCGGTGCAGCCTTTT
T-EraP-pro-Ichl-R	ATTGACTATCCGGTATTACCCGGCATGACAGGAGTAAAACGCG CGCGCAATTGAAAAAT
**RT-qPCR analysis**	
qPCR-Eracas3-1-F	GCGGCTATATCTACGGCAACAC
qPCR-Eracas3-1-R	GGTTCTGCATCATCGGCTTCG
qPCR-Eracse1-2-F	AGCTGCTGATCTGCCTGTTACA
qPCR-Eracse1-2-R	GCTGCTGCGGATGGTTGAGA
qPCR-Eracse2-3-F	GCCTAGCGAAGATAAGCAGCAA
qPCR-Eracse2-3-R	CACCTCGGGTTCGGCATGAATA
qPCR-Eracas7-4-F	CTGATGAGCGAGGTGGGTAACG
qPCR-Eracas7-4-R	AGAACTCCTGGGTGCCAAGATG
qPCR-Eracas5-5-F	GCAGCATCAGGCGGAACATG
qPCR-Eracas5-5-R	AACTGGCGTGGTAGGTGGATT
qPCR-Eracas6-6-F	AAGCAGGCGGTGGCAGGAAA
qPCR-Eracas6-6-R	AGGCACGGGCGATGGATAACA
qPCR-Eracas1-7-F	AATGGCGTGGGCGTCGTTAC
qPCR-Eracas1-7-R	GCGGCTTGCCACTGTGAACA

qPCR-Eracas2-8-F	ATGGCTGCTGGAAGTGCGTG
qPCR-Eracas2-8-R	AAACCCGACTCGCTGTTAGTGG
16sRNA-F	ACCCTTATCCTTTGTTGCC
16sRNA-R	TATGAGGTCCGCTTGCTCT
**Construction of the strain ActSY01**	
T-Pcas3-aprpBAD-F	TTTGGCTTAAAAAGGGAATGTGGGTTACACGAAGGGTAACTCG TTCTCCGCTCATGAGC
T-Pcas3-aprpBAD-R	TTTTCCCCAGTAATGGCATATATATTTAAAAGGTTCCATTTTTT ATAACCTCCTTAGAG
T-Pcas-kan23119-1-F	GAAGGGATGACCAGAGTCATCCCTGCAAATCCCAAATAAGGC GTCCCGGAAAACGATTC
T-Pcas-kan23119-1-R	TAGTAGCTAGCATTATACCTAGGACTGAGCTAGCTGTCAACAC GCTGCCGCAAGCACTCA
T-Pcas-kan23119-2-F	AGGTATAATGCTAGCTACTAGAGAAAGAGGAGAAATACTAGA TGAATTTGCTTATTGATA
T-Pcas-kan23119-2-R	CCAACAGTTTTTCCATTGGA
T-Pcas-kan23119-2-II-R	CGGGCGTACAGGGATCCAGT
**Constructions of the *recA*-deficient knock-out mutants**	
ΔrecA-FRT-MG1655-F	GACTATCCGGTATTACCCGGCATGACAGGAGTAAAAATGATTC CGGGGATCCGTCGACC
ΔrecA-FRT-MG1655-R	GCCGCAGATGCGACCCTTGTGTATCAAACAAGACGATTATGTA GGCTGGAGCTGCTTC
ΔrecA-FRT-MDS42-F	ATGGCTATCGACGAAAACAAACAGAAAGCGTTGGCGGCAATT CCGGGGATCCGTCGACC
ΔrecA-FRT-MDS42-R	TTAAAAATCTTCGTTAGTTTCTGCTACGCCTTCGCTATCTGTAG GCTGGAGCTGCTTC
**Constructions of CRISPR interference plasmids in the ISs trapping system**	
Tar-tet-F	ATCAGGTTTGTGCCAATACCAGTAGAAACAGACGAAGAATTG GCCAGAAAAAAAAGAATATATAAG
Tar-tet-R	GCGCAACGCAATTAATGTAAGTTAGCGCGAATTGATCTGGTGG CCACGCCGGCTTCCATTCAGGTC
Tar-SmR-pTrc-1-F	GCACCCTCGCAAGCTCGGTTGCGGCCGCAATCGGGCAAATCTC GAGTTCATGTGCAGCTCCATAA
Tar-SmR-pTrc-1-R	TAATTGTCAACAGCTCATTTAGATCTCTCGAGACTGGCGAGCG GCATCTTAT
Tar-SmR-pTrc-2-F	ATAAGATGCCGCTCGCCAGTCTCGAGAGATCTAAATGAGCTGT TGACAATTA
Tar-SmR-pTrc-2-R	TTGTGCCGATATCTAAGCCTATTGAGTATTTCTTATCCATAGAT CCTTTCTCCTCTTTGC
SpCas9-HF1-1-F	TTCGAATTCCTTATTATGTT
SpCas9-HF1-1-R	GAAGATTTTTATCAAACGCTGTCATGCGTTCAATA
SpCas9-HF1-2-F	TATTGAACGCATGACAGCGTTTGATAAAAATCTTC
SpCas9-HF1-2-R	TCAATTTTCGAGACAACGCTCCCCAACCAGTATAA
SpCas9-HF1-3-F	TTATACTGGTTGGGGAGCGTTGTCTCGAAAATTGA

SpCas9-HF1-3-R	TATCATCATGGATCAGCGCCATAAAATTGCGATTG
SpCas9-HF1-4-F	CAATCGCAATTTTATGGCGCTGATCCATGATGATA
SpCas9-HF1-4-R	CCACATGCTTAGTGATCGCGCGAGTTTCAACCAAT
SpCas9-HF1-5-F	ATTGGTTGAAACTCGCGCGATCACTAAGCATGTGG
SpCas9-HF1-5-R	TCTAGACGATCGTTAAGCAGCCAGAGCGTAGTTTTCGTCGTTAGCAGCGTCACCTCCTAGCTGACT
Tar-Δλ-red-kan-F	TATGAAACACGCATTGATTTGAGTCAGCTAGGAGGTGACTAAGGCGTCCCGGAAAACGATTC
Tar-Δλ-red-kan-R	TTGCGCCTACCCGGATATTATCGTGAGGATGCGTACGTACACGCTGCCGCAAGCACTCA
Tar-Δλ-red-kan-ssrA-F	GCGCACATTTCCCCGAAAAGTGCCACCTGCATCGATTTAGGCGTCCCGGAAAACGATTC
Tar-Δλ-red-kan-ssrA-R	TTGCGCCTACCCGGATATTATCGTGAGGATGCGTACGTACACGCTGCCGCAAGCACTCA
Tar-spe-FnCpf1-1-F	AGCGCAATGACATTCTTGCAGGTATCTTCGAGCCAGCCACGATCGACATTGATCTGGCT
Tar-spe-FnCpf1-1-R	AATTCTTGATAAATTGACATAGATCCTTTCTCCTCTTTGC
Tar-spe-FnCpf1-2-F	GCAAAGAGGAGAAAGGATCTATGTCAATTTATCAAGAATT
Tar-spe-FnCpf1-2-R	TTCTAAATACATTCAAATATGTATCCGCTCATGTCTAGACGATCGTTAGTTATTCCTAT
Tar-spe-SpCas9-HF1(D10A)-1-F	CAGCGCAATGACATTCTTGC
Tar-spe-SpCas9-HF1(D10A)-1-R	CTATTTGTGCCGATAGCTAAGCCTATTGAGT
Tar-spe-SpCas9-HF1(D10A)-2-F	ACTCAATAGGCTTAGCTATCGGCACAAATAG
Tar-spe-SpCas9-HF1(D10A)-2-R	CTGACCGCTTCCTCGTGCTT
Tar-spe-SpCas9-HF1(H840A)-1-F	CAGCGCAATGACATTCTTGC
Tar-spe-SpCas9-HF1(H840A)-1-R	CTTTGTGGAACAATGGCATCGACATCATAATC
Tar-spe-SpCas9-HF1(H840A)-2-F	GATTATGATGTCGATGCCATTGTTCCACAAAG
Tar-spe-SpCas9-HF1(H840A)-2-R	CTGACCGCTTCCTCGTGCTT
Tar-spe-F	CAGCGCAATGACATTCTTGC
Tar-spe-CasOP01-R	ATGCTATACTTTTTATCCATAGATCCTTTCTCCTCTTTGC
Tar-spe-CasOP01-1-F	GCAAAGAGGAGAAAGGATCTATGGATAAAAAGTATAGCAT
Tar-spe-CasOP01-1-R	CGGGTCATCCAGGCGAAGCGACTGTTCCCGCGAGCCAGAG
Tar-spe-CasOP01-2-F	CTCTGGCTCGCGGGAACAGTCGCTTCGCCTGGATGACCCG
Tar-spe-CasOP01-2-R	ATTGCACGGGTCTCTACCAGCTGACGTTTGATAAAACCCG
Tar-spe-CasOP01-3-F	CGGGTTTTATCAAACGTCAGCTGGTAGAGACCCGTGCAAT
Tar-spe-CasOP01-3-R	TTTCTAAATACATTCAAATATGTATCCGCTCATGTCTAGACGATCGTCAGTCTCCCCCCAGCTGACTCA
Tar-spe-CasOP01-ssrA-3-R	TTTCTAAATACATTCAAATATGTATCCGCTCATGTCTAGACGATCGTCAGGCGGCCAGGGCGTAGT
Tar-spe-Cpf1OP01-R	AATTCCTGGTAAATCGACATAGATCCTTTCTCCTCTTTGC
Tar-spe-FnCpf1OP01-1-F	GCAAAGAGGAGAAAGGATCTATGTCGATTTACCAGGAATT
Tar-spe-FnCpf1OP01-1-R	TCAGTTCCTGTTCTTTTTTCGAAGGATTATCGAGATTTTT
Tar-spe-FnCpf1OP01-2-F	AAAAATCTCGATAATCCTTCGAAAAAAGAACAGGAACTGA
Tar-spe-FnCpf1OP01-2-R	CGTAAAGCGCTTATCCTTGATCAGATCATATTCAAACACG

Tar-spe-FnCpf1OP01-3-F	CGTGTTTGAATATGATCTGATCAAGGATAAGCGCTTTACG
Tar-spe-FnCpf1OP01-3-R	TTTCTAAATACATTCAAATATGTATCCGCTCATGTCTAGACGAT CGTCAATTATTACGGTTCTGGA
Tar-spe-CasOP02-1-R	GCCACCATCGATGTAGCCCGCATATCCGTTTTTTGACTG
Tar-spe-CasOP02-2-F	CAGTCAAAAAACGGATATGCGGGCTACATCGATGGTGGC
Tar-spe-CasOP02-2-R	TTTACGGGTCATCCAGGCAAACCTACTGTTCCCGCGAGC
Tar-spe-CasOP02-3-F	GCTCGCGGGAACAGTAGGTTTGCCTGGATGACCCGTAAA
Tar-spe-CasOP02-3-R	TCGTATCGGTTTGTCCCGGTGTTTATTATACGCTGAAAGCAC
Tar-spe-CasOP02-4-F	GTGCTTTCAGCGTATAATAAACACCGGGACAAACCGATACGA
Tar-spe-III-R	GTGGCACTTTTCGGGGAAAT
Tar-spe-FnCpf1OP02-1-R	ATTACGGATGCGCAGAATGTCTTCAGACGGA
Tar-spe-FnCpf1OP02-2-F	TCCGTCTGAAGACATTCTGCGCATCCGTAAT
Tar-spe-CasOP03-1-R	CGACTCTTCCAGGCGGTGAAAAAAGCTATCATCTACCTTCGC
Tar-spe-CasOP03-2-F	TTCACCGCCTGGAAGAGTCGTTTCTGGTTGAAGAGGATAAAAA ACACGAACGCCAT
Tar-spe-CasOP03-2-R	GTATTTCTCATGGTACGCCACCTCGTCCACTATATTA
Tar-spe-CasOP03-3-F	TAATATAGTGGACGAGGTGGCGTACCATGAGAAATAC
Tar-spe-CasOP03-3-R	GATTGATCCATTATCGAATGTTCGCTGTTTACGCAGC
Tar-spe-CasOP03-4-F	GCTGCGTAAACAGCGAACATTCGATAATGGATCAATC
Tar-spe-CasOP03-4-R	GTGTTCTTTCAGAATTTGGCTCCCCAGTTCT
Tar-spe-CasOP03-5-F	AGAACTGGGGAGCCAAATTCTGAAAGAACAC
Tar-spe-FnCpf1OP03-1-R	AAAGAGATACAATTTACCTTGGTTCACCACC
Tar-spe-FnCpf1OP03-2-F	GGTGGTGAACCAAGGTAAATTGTATCTCTTT
Tar-spe-FnCpf1OP03-2-R	TGCCTTTTCTTTCAGTAAGAGGTTGATTTCATC
Tar-spe-FnCpf1OP03-3-F	GATGAAATCAACCTCTTACTGAAAGAAAAGGCA
**Constructions of NHEJ expression plasmids**	
pBAD-Mm-ku-1-F	GCGGCCGCCGATCGTCTAGACATGAGCG
pBAD-Mm-ku-1-R	CCCTTCCAGATGGAGCGCATTTTTTATAACCTCCTTAGAG
pBAD-Mm-ku-2-F	CTCTAAGGAGGTTATAAAAAATGCGCTCCATCTGGAAGGG
pBAD-Mm-ku-2-R	ACTAGTTGCTTGGATTCTCACCAATA
pBAD-Ms-ku-1-F	ACATGTCGATCGTCTAGACATGAGCG
pBAD-Ms-ku-1-R	CCCTTCCAGATGGATCGCATTTTTTATAACCTCCTTAGAG
pBAD-Ms-ku-2-F	CTCTAAGGAGGTTATAAAAAATGCGATCCATCTGGAAGGG
pBAD-Ms-ku-2-R	ACTAGTTGCTTGGATTCTCACCAATA
pBAD-Bs-ku-1-F	GCGGCCGCCGATCGTCTAGACATGAGCG
pBAD-Bs-ku-1-R	AGAGAAGGAGTACGATTCATTTTTTATAACCTCCTTAGAG
pBAD-Bs-ku-2-F	CTCTAAGGAGGTTATAAAAAATGAATCGTACTCCTTCTCT
pBAD-Bs-ku-2-R	ACTAGTTGCTTGGATTCTCACCAATA
**Determination of the indel mutations in the gene *ompF***	
V-ompF-F	GTGCGAGCACGTTTGTCATT
V-ompF-R	CTTCGTTGGTCGTGTTGGCG
**Detection of the target site duplications**	
S-IS1-1-F	CGCCATTCATGGCCATATCA

S-IS1-2-F	CCTGAAGGGAAAGCTGCACG
S-IS2-1-F	CCTGTATGTGCCCGTTTCGA
S-IS2-2-F	CCGGAGAGTAACGGAATAGC
S-IS3-1-F	CCGATGCGTTCAGCAAGCTT
S-IS3-2-F	GAACACTTTATCAGCCGGGA
S-IS5-1-F	GTACCAATGCTGCATGCAGT
S-IS5-2-F	ATACGTCAGTGGGAGAGATC
S-IS10-1-F	TTGGTTGGCAGGTTACGGCC
S-IS10-2-F	GCTCAGAAACAAGGTTGGGA
S-IS150-1-F	GAGCGCGCGTAAACCAGCTT
S-IS150-2-F	CTGTTCTGCACTCTGACCAG
**PCR amplifications of the complete coding regions of SpCas9-HF1, FnCpf1 and their derivatives**	
S-univer-c&9-F	TAAGATGCCGCTCGCCAGTC
S-univer-c&9-R	CTGACCGCTTCCTCGTGCTT
**Sanger sequencing for the complete coding regions of SpCas9-HF1, FnCpf1 and their derivatives**	
S-SpCas9-HF1-1M-F	GCTGGTTTTCGCATTCCTTC
S-SpCas9-HF1-2-F	CAAATTCACTTGGGTGAGCT
S-SpCas9-HF1-3-F	CGCCAAGTTAATCACTCAAC
S-SpCas9-HF1-4-F	GGTAATCACTTTAACCTCTC
S-FnCpf1-1-F	CCCACAGGGTAAAACACTTG
S-FnCpf1-2-F	GGCAGAAGAGCTAACCTTTG
S-FnCpf1-3-F	GCAAACTTTGCGGCTATTCC
S-FnCpf1-4-F	TAAGCATCCGGAGTGGAAAG
S-FnCpf1-5-F	CTCAAAATAGCCCTTATCAAGG
S-SpCas9-HF1-OP01-1-F	CTTTTCATCCAGCTGGTGCA
S-SpCas9-HF1-OP01-2-F	CCGCACCAGATTCACTTGGG
S-SpCas9-HF1-OP01-3-F	GTACGCCCATCTGTTTGATG
S-SpCas9-HF1-OP01-4-F	CCCGCAGTGACAAAAACCGT
S-SpCas9-HF1-OP01-5-F	TGTGGGATAAAGGTCGGGAC
S-FnCpf1-OP01-1-F	GCAGCAATGATATTCCGACC
S-FnCpf1-OP01-2-F	CCGCGGTGTTGGAATACATT
S-FnCpf1-OP01-3-F	CGGCGTTATGAACAAGAAAA
S-FnCpf1-OP01-4-F	CCCGAAAAAGGAAAGCGTGT
**Sanger sequencing for the sgRNAs and crRNAs expression cassettes**	
V-p15A-cm-circle-F	CAAGGCGACAAGGTGCTGAT
V-p15A-cm-circle-R	GGTAGCTCAGAGAACCTTCGA

Table S2. Plasmids used in this study.

Plasmids	Descriptions	Source
**General plasmids**		
pBluescript SK(+)	the vector backbone for cloning PCR products	Stratagene
p15A-cm	the verctor backbone for cloning both sgRNAs and crRNAs expression cassettes	1
pUC57-1.8K	the vector backbone for cloning NHEJ expression cassettes	Qingke[a], General[b]
pClone007	the vector backbone for cloning the type I-E CRISPR Cas system of *Erwinia amylovora* 7-3	Qingke[a]
pSET152	the template plasmid for amplifying the apramycin selective marker	2
**the plasmids associated with the type I-E CRISPR-Cas system of *Erwinia amylovora* 7-3**		
pClone007-EraCas01	the plasmid pClone007 containing the fragment EraCas01	This study
pClone007-EraCas02	the plasmid pClone007 containing the fragment EraCas02	This study
pClone007-EraCas03	the plasmid pClone007 containing the fragment EraCas03	This study
pClone007-EraCas04	the plasmid pClone007 containing the fragment EraCas04	This study
pClone007-EraCas05	the plasmid pClone007 containing the fragment EraCas05	This study
pEraCas234	the plasmid pClone007 containing the fragment EraCas02, EraCas03 and EraCas04	This study
pEraCas1234	the plasmid pClone007 containing the fragment EraCas01, EraCas02, EraCas03 and EraCas04	This study
pEraCas	the plasmid pClone007 containing the engineered type I-E CRISPR-Cas locus of *E. amylovora* 7-3	This study
pEraCas-IS10-Apr	derived from the plasmid pEraCas, in which the IS10 element together with the apramycin resistance marker were inserted into the coding region of *cas7*	This study
pEraCas-IS10	derived from the plasmid pEraCas-IS10-Apr, in which the apramycin resistance marker was removed	This study
p15A-Eraprotos	the plasmid p15A-cm containing the engineered protospacers that were designed from the plasmid p7-3	This study
SK(+)-spe	the plasmid pBluescript SK(+) containing the spectinomycin resistance cassette	This study
p15A-Eraprotos-spe	the plasmid p15A-Eraprotos with the insertion of the spectinomycin resistance cassette	This study
p15A-spe	derived from the plasmid p15A-Eraprotos-spe, in which all protospacers were deleted	This study
pEraCas-pBAD	derived from the plasmid pEraCas, in which the *araC* gene and pBAD promoter were inserted in front of *cas3* to dirve the expression of *cas* operon in the presence of L-arabinose	This study

**the plasmids associated with the type I-E CRISPR/Cas system of *E. coli* DH10B and ActSY01**		
p15A-sgRNA::lacZ02	the sgRNA::lacZ02 expression cassette was cloned into plasmid p15A-cm	This study
p15A-CRISPR::arpA	the CRISPR array expression cassette (CRISPR::arpA) was cloned into plasmid p15A-cm	This study
p15A-CRISPR::ftsA	the CRISPR array expression cassette (CRISPR::ftsA) was cloned into plasmid p15A-cm	This study
**CRISPR interference plasmids in the ISs trapping system**		
pCas	the original vector backbone for the constructions of the CRISPR interference plasmids	Addgene # 62225
pY001	the template plasmid containing the complete coding region of FnCpf1	Addgene # 69973
pCas-tet	pCas with the insertion of tetracycline resistance marker	This study
pCasY-1	derived from the plasmid pCas-tet, in which kanamycin resistance marker together with its neighboring sequences were substituted with both spectinomycin resistance marker and pTrc promoter	This study
pCasY-2	derived from the plasmid pCasY-1, in which the complete coding region of SpCas9 was replaced with the high-fidelity SpCas9-HF1 nuclease fused with the ssrA degratation tag to its C-terminus	This study
pCasY-3-ΔSIM-kan	derived from the plasmid pCasY-2, in which the λ-red expression cassette together with the ssrA degratation tag were substituted with the kanamycin resistance marker	This study
pCasY-3-ΔSIM-kan (D10A)	pCasY-3-ΔSIM-kan with the mutation D10A in the coding region of the SpCas9-HF1	This study
pCasY-3-ΔSIM-kan (H840A)	pCasY-3-ΔSIM-kan with the mutation H840A in the coding region of the SpCas9-HF1	This study
pCasY-3-ΔSIM-kan (D10A&H840A)	pCasY-3-ΔSIM-kan with the mutations D10A and H840A in the coding region of the SpCas9-HF1	This study
pCasY-3-ΔSIM-kan-ssrA-PvuI	the vector backbone derived from the plasmid pCasY-3-ΔSIM-kan-ssrA, in which both the partial C-terminus of spectinomycin resistance protein coding region and the complete SpCas9-HF1-ssrA expression cassette were deleted	This study
pCasY-3-ΔSIM-kan-OP01	the vector backbone pCasY-3-ΔSIM-kan-ssrA-PvuI with the insertions of both C-terminus of spectinomycin resistance protein coding region and the SpCas9-HF1-OP01 expression cassette	This study
pCasY-3-ΔSIM-kan-OP02	the vector backbone pCasY-3-ΔSIM-kan-ssrA-PvuI with the insertions of both C-terminus of spectinomycin resistance protein coding region and the SpCas9-HF1-OP02 expression cassette	This study

pCasY-3-ΔSIM-kan-OP03	the vector backbone pCasY-3-ΔSIM-kan-ssrA-PvuI with the insertions of both C-terminus of spectinomycin resistance protein coding region and the SpCas9-HF1-OP03 expression cassette	This study
pCasY-3-ΔSIM-kan-ssrA	derived from the plasmid pCasY-2, in which the λ-red expression cassette was substituted with the kanamycin resistance marker	This study
pCasY-3-ΔSIM-kan-OP01-ssrA	the vector backbone pCasY-3-ΔSIM-kan-ssrA-PvuI with the insertions of both C-terminus of spectinomycin resistance protein coding region and the SpCas9-HF1-OP01-ssrA expression cassette	This study
pCasY-3-ΔSIM-kan-OP02-ssrA	the vector backbone pCasY-3-ΔSIM-kan-ssrA-PvuI with the insertions of both C-terminus of spectinomycin resistance protein coding region and the SpCas9-HF1-OP02-ssrA expression cassette	This study
pCasY-3-ΔSIM-kan-OP03-ssrA	the vector backbone pCasY-3-ΔSIM-kan-ssrA-PvuI with the insertions of both C-terminus of spectinomycin resistance protein coding region and the SpCas9-HF1-OP03-ssrA expression cassette	This study
pCpf1Y-3-ΔSIM-kan	the vector backbone pCasY-3-ΔSIM-kan-ssrA-PvuI with the insertions of both C-terminus of spectinomycin resistance protein coding region and the FnCpf1 expression cassette	This study
pCpf1Y-3-ΔSIM-kan-OP01	the vector backbone pCasY-3-ΔSIM-kan-ssrA-PvuI with the insertions of both C-terminus of spectinomycin resistance protein coding region and the FnCpf1-OP01 expression cassette	This study
pCpf1Y-3-ΔSIM-kan-OP02	the vector backbone pCasY-3-ΔSIM-kan-ssrA-PvuI with the insertions of both C-terminus of spectinomycin resistance protein coding region and the FnCpf1-OP02 expression cassette	This study
pCpf1Y-3-ΔSIM-kan-OP03	the vector backbone pCasY-3-ΔSIM-kan-ssrA-PvuI with the insertions of both C-terminus of spectinomycin resistance protein coding region and the FnCpf1-OP03 expression cassette	This study
**sgRNAs/crRNAs expression plasmids**		
p15A-cm-sgRNA-nonespacer	the sgRNA cassette without any spacer sequence cloned into plasmid p15A-cm	This study
p15A-cm-sgRNA::ompF	the sgRNA::ompF expression cassette was cloned into plasmid p15A-cm	This study
p15A-cm-sgRNA::pyrF	the sgRNA::pyrF expression cassette was cloned into plasmid p15A-cm	This study
p15A-cm-sgRNA::lpp	the sgRNA::lpp expression cassette was cloned into plasmid p15A-cm	This study

p15A-cm-sgRNA::lacZ	the sgRNA::lacZ expression cassette was cloned into plasmid p15A-cm	This study
p15A-cm-sgRNA::ompF pair 01	the sgRNA::ompF pair 01 expression cassette was cloned into plasmid p15A-cm	This study
p15A-cm-sgRNA::ompF pair 02	the sgRNA::ompF pair 02 expression cassette was cloned into plasmid p15A-cm	This study
p15A-cm-crRNA-nonespacer	the crRNA cassette without any spacer sequence cloned into plasmid p15A-cm	This study
p15A-cm-crRNA::ompF	the crRNA::ompF expression cassette was cloned into plasmid p15A-cm	This study
p15A-cm-crRNA::pyrF	the crRNA::pyrF expression cassette was cloned into plasmid p15A-cm	This study
p15A-cm-crRNA::lpp	the crRNA::lpp expression cassette was cloned into plasmid p15A-cm	This study
p15A-cm-crRNA::lacZ	the crRNA::lacZ expression cassette was cloned into plasmid p15A-cm	This study
**NHEJ expression plasmids**		
pUC57-1.8K-Bs-ku	the complete coding region of the Ku from the *Bacillus subtilis* 168 was cloned into the plasmid pUC57-1.8K	This study
SK(+)-pBAD-Bs-ku	the gene *ku* was amplified from the plasmid pUC57-1.8K-Bs-ku and fused together with the promoter pBAD by overlap PCR, subsequently, the overlap PCR product was cloned into the plasmid pBluescript SK(+)	This study
pUC57-1.8K-Bs-ligD	the gene *ligD* from the *Bacillus subtilis* 168, was assembled with the promoter J23119, and then cloned into the plasmid pUC57-1.8K	This study
pUC57-1.8K-Ms-ku	the complete coding region of the Ku from the *Mycobacterium smegmatis* mc^2155 was cloned into the plasmid pUC57-1.8K	This study
SK(+)-pBAD-Ms-ku	the gene *ku* was amplified from the plasmid pUC57-1.8K-Ms-ku and fused together with the promoter pBAD by overlap PCR, subsequently, the overlap PCR product was cloned into the plasmid pBluescript SK(+)	This study
pUC57-1.8K-Ms-ligD	the gene *ligD* from the *Mycobacterium smegmatis* mc^2155, was assembled with the promoter J23119, and then cloned into the plasmid pUC57-1.8K	This study
pUC57-1.8K-Mm-ku	the complete coding region of the Ku from the *Mycobacterium marinum* M was cloned into the plasmid pUC57-1.8K	This study

SK(+)-pBAD-Mm-ku	the gene *ku* was amplified from the plasmid pUC57-1.8K-Mm-ku and fused together with the promoter pBAD by overlap PCR, subsequently, the overlap PCR product was cloned into the plasmid pBluescript SK(+)	This study
pUC57-1.8K-Mm-ligD	the gene *ligD* from the *Mycobacterium marinum* M, was assembled with the promoter J23119, and then cloned into the plasmid pUC57-1.8K	This study
pBs-NHEJ	the DNA cassette harboring the gene *ku* and pBAD promoter was digested from the plasmid SK(+)-pBAD-Bs-ku, and then cloned into the plamid pUC57-1.8K-Bs-ligD	This study
pMs-NHEJ	the DNA cassette harboring the gene *ku* and pBAD promoter was digested from the plasmid SK(+)-pBAD-Ms-ku, and then cloned into the plamid pUC57-1.8K-Ms-ligD	This study
pMm-NHEJ	the DNA cassette harboring the gene *ku* and pBAD promoter was digested from the plasmid SK(+)-pBAD-Mm-ku, and then cloned into the plamid pUC57-1.8K-Mm-ligD	This study

[a] denoted the Beijing Qingke Biotechnology Co., Ltd.

[b] indicated the General Biological System (Anhui) Co., Ltd (Anhui, China).

Table S3. Strains used in this study.

Strains	Descriptions	Source
Escherichia coli		
DH10B	str. K-12 F^-	Invitrogen
MG1655	K-12 F^- λ^- *ilvG⁻ rfb-50 rph-1*	[3]
MG1655-Δ*recA*	MG1655 strain with *recA* gene disrupted	This study
MDS42	a derivative of *E. coli* K-12 without IS elements	[4]
MDS42-Δ*recA*	MDS42 strain with *recA* gene disrupted	This study
GB08-red	for expression of λ-red recombination system	[1]
SYHY01	DH10B equipped with an exogenous *E. amylovora* type I-E CRISPR-Cas system in a single plasmid pEraCas	This study
SYHY02	DH10B harboring an exogenous *E. amylovora* type I-E IS10-aborted CRISPR-Cas system in a single plasmid pEraCas-IS10	This study
SYHY01::p15A-Eraprotos-spe	SYHY01 bearing plasmid p15A-Eraprotos-spe	This study
SYHY02::p15A-Eraprotos-spe	SYHY02 bearing plasmid p15A-Eraprotos-spe	This study
SYHY01::p15A-spe	SYHY01 bearing plasmid p15A-spe	This study
SYHY02::p15A-spe	SYHY02 bearing plasmid p15A-spe	This study
SYHY03	DH10B containing all protospacers that were designed from plasmid p7-3	This study
DH10B::pEraCas	DH10B containing plasmid pEraCas	This study
DH10B::pEraCas-pBAD	DH10B containing plasmid pEraCas-pBAD	This study
ActSY01	DH10B harboring an engineered type I-E CRISPR/Cas system	This study

Table S4. Analysis of a total of 19 larger amplicons containing an IS element insertion into *cas* genes within the type I-E CRISPR/Cas system of the strain ActSY01.

Samples	IS type	TSD sequences	TSD length
A01	IS10	CGCAGAACA	9
A02	IS10	TGCTATGCT	9
A03	IS10	TGTCAGGCA	9
A04	IS10	TGCTATGCT	9
A05	IS5	TTAG	4
A06	IS1	ACGCCAGCG	9
A07	IS1	GGCAATGGC	9
A09	IS2	GGCAC	5
A10	IS10	TGCTATGCT	9
A11	IS10	GGCCTGGCT	9
A12	IS10	CACTCGACG	9
A13	IS5	TTAA	4
A14	IS10	CACTCGACG	9
A16	IS10	TGCTGCGCA	9
A17	IS10	CACTCGACG	9
A18	IS1	CTGGTTTCC	9
A19	IS10	GGCCTGGCT	9
A20	IS10	AGCTTCGCC	9
A23	IS10	CGCAGAACA	9

Table S5. Analysis of a total of 44 larger amplicons containing an IS element insertion into the coding region of SpCas9-HF1.

Samples	IS type	TSD sequences	TSD length
1108-02A1	IS1	GAGATGGCG	9
1108-02A2	IS1	GATTTGCGC	9
1108-02A3	IS1	GAGATGGCG	9
1108-02A5	IS5	CAAG	4
1108-02B4	IS1	GTAACCGTT	9
1110-03A1	IS5	CTAA	4
1110-03A2	IS10	TGCTAAGTC	9
1110-03A3	IS10	TGCTAAGTC	9
1110-03A4	IS10	CACTCAACG	9
1110-03A5	IS1	GTGATTATG	9
1110-03A6	IS10	TGCTAAGTC	9
1110-03A7	IS10	CACTCAACG	9
1110-03A8	IS3	GGT	3
1110-03B1	IS10	CACTCAACG	9
1110-03B2	IS10	TGCTAAGTC	9
1110-03B3	IS10	TGCTAAGTC	9
1110-03B4	IS10	TGCCCCAAG	9
1110-03B5	IS1	AGTAACCGT	9
1110-03B6	IS10	TGCTAAGTC	9
1110-03B7	IS10	CACTCAACG	9
1110-03B8	IS10	TGCTAAGTC	9
1110-03C1	IS1	CAAGGTTC	8
1110-03C2	IS5	TTAG	4
1110-03C3	IS10	TGCTAAGTC	9
1110-03C5	IS10	CACTCAACG	9
1110-03C6	IS1	CAAGGTTC	8
1110-03C7	IS10	TGCTAAGTC	9
1110-03C8	IS10	CGCATGACA	9
1110-03D1	IS10	TGCTAAGTC	9
1110-03D3	IS3	GGT	3
1110-03D4	IS3	GGT	3
1110-03D5	IS10	TGCTAAGTC	9
1110-03D6	IS10	TGCTAAGTC	9
1110-03D7	IS1	CAAGGTTC	8
1110-03D8	IS10	CACTCAACG	9
1110-03E4	IS10	CACTCAACG	9
1110-03E5	IS3	GGT	3
1110-03E6	IS10	CACTCAACG	9
1110-03E8	IS1	GTAACCGTT	9

1110-03F1	IS5	CTAA	4
1110-03F2	IS10	AGCTCTATC	9
1110-03F3	IS10	TGCTAAGTC	9
1110-03F4	IS10	CACTCAACG	9
1110-03F5	IS10	CACTCAACG	9

Table S6. Summary of whole-genome sequencing of 11 CRISPR-tolerant mutants and the control strain.

Samples	Genome accession	IS type[a]
SY221	CP106998	/
SY22A1	CP107005	IS10
SY22A3	CP107004	IS1
SY22A4	CP106999	IS5
SY22A6	CP106994	IS1
SY22B1	CP107003	IS5
SY22B2	CP107000	/
SY22B3	CP106995	IS10
SY22B5	CP107001	IS2
SY22B6	CP106997	/
SY22C3	CP107002	IS2
SY22D8	CP106996	/

[a] identified by PCR and Sanger sequencing.

"/" indicated that no IS element was inserted into the coding region of SpCas9-HF1 in the given sample.

Table S7. The non-canonical length of TSD sequences generated by IS1 in our CRISPR/Cas-mediated ISs trapping system.

Samples	TSD sequences	TSD length
0201-2F3	ATCAAGACGAT	11
0310-3A3	CAAATATACAACAAGGACTTTTC	23
0310-3D1	CAAATATACAACAAGGACTTTTC	23
0310-3E6	CAAATATACAACAAGGACTTTTC	23
0526-1B5	GTTTTAT	7

Supplementary References

1. Wang, H. *et al.* ExoCET: exonuclease in vitro assembly combined with RecET recombination for highly efficient direct DNA cloning from complex genomes. *Nucleic Acids Res* **46**, e28 (2018).
2. Bierman, M. *et al.* Plasmid cloning vectors for the conjugal transfer of DNA from *Escherichia coli* to *Streptomyces* spp. *Gene* **116**, 43-49 (1992).
3. Blattner, F.R. *et al.* The complete genome sequence of *Escherichia coli* K-12. *Science* **277**, 1453-1462 (1997).
4. Pósfai, G. *et al.* Emergent properties of reduced-genome *Escherichia coli*. *Science* **312**, 1044-1046 (2006).
