# Supplementary material for: Insertion sequence transposition inactivates CRISPR-Cas immunity
Source: Nat Commun. 2023 Jul 20;14:4366. doi: 10.1038/s41467-023-39964-7 (PMC10359306; doi:10.1038/s41467-023-39964-7)
Supplement: Supplementary file 3 — Description of Additional Supplementary Files [file 41467_2023_39964_MOESM3_ESM.pdf]

Description of Additional Supplementary Files:

Supplementary Data 1. The multiple correspondence analysis of 386 different plasmids which may be targeted by the CRISPR-Cas system in five of the strains containing ISs in their cas genes. Numbers inside the table represent the GenBank accession numbers.

Supplementary Data 2. Taxonomic analysis of 163 cases of ISs transpositions into cas genes.

Supplementary Data 3. The sample size information for supplementary figure 16.

Supplementary Data 4. The sample size information for supplementary figure 17.

Supplementary Data 5. The sample size information for supplementary figure 19.

Supplementary Data 6. The sample size information for supplementary figure 21.
