# Supplementary material for: Insertion sequence transposition inactivates CRISPR-Cas immunity
Source: Nat Commun. 2023 Jul 20;14:4366. doi: 10.1038/s41467-023-39964-7 (PMC10359306; doi:10.1038/s41467-023-39964-7)
Supplement: Supplementary file 10 — Reporting Summary [file 41467_2023_39964_MOESM10_ESM.pdf]

nature portfolio

Corresponding author(s): Qianjin Kang

Last updated by author(s): Jun 28, 2023

Reporting Summary

Nature Portfolio wishes to improve the reproducibility of the work that we publish. This form provides structure for consistency and transparency in reporting. For further information on Nature Portfolio policies, see our Editorial Policies and the Editorial Policy Checklist.

Statistics

For all statistical analyses, confirm that the following items are present in the figure legend, table legend, main text, or Methods section.

n/a	Confirmed	
☐	☒	The exact sample size (*n*) for each experimental group/condition, given as a discrete number and unit of measurement
☐	☒	A statement on whether measurements were taken from distinct samples or whether the same sample was measured repeatedly
☐	☒	The statistical test(s) used AND whether they are one- or two-sided<br>*Only common tests should be described solely by name; describe more complex techniques in the Methods section.*
☒	☐	A description of all covariates tested
☐	☒	A description of any assumptions or corrections, such as tests of normality and adjustment for multiple comparisons
☐	☒	A full description of the statistical parameters including central tendency (e.g. means) or other basic estimates (e.g. regression coefficient) AND variation (e.g. standard deviation) or associated estimates of uncertainty (e.g. confidence intervals)
☐	☒	For null hypothesis testing, the test statistic (e.g. *F*, *t*, *r*) with confidence intervals, effect sizes, degrees of freedom and *P* value noted<br>*Give P values as exact values whenever suitable.*
☒	☐	For Bayesian analysis, information on the choice of priors and Markov chain Monte Carlo settings
☒	☐	For hierarchical and complex designs, identification of the appropriate level for tests and full reporting of outcomes
☐	☒	Estimates of effect sizes (e.g. Cohen's *d*, Pearson's *r*), indicating how they were calculated

Our web collection on statistics for biologists contains articles on many of the points above.

Software and code

Policy information about availability of computer code

Data collection	No software was used for data collection.
Data analysis	Genome sequences were downloaded using ncbi-genome-download scripts (https://github.com/kblin/ncbi-genome-download/) (version 0.3.1) and the executable software Aspera (IBM Aspera CLI version 3.9.6.1467.159c5b1, ascp version 3.9.3.177167). Both sgRNAs (for SpCas9-HF1) and crRNAs (for FnCpf1) were designed using the CRISPOR algorithm (version 5.01). Two pairs of sgRNAs with different offsets were designed by the CHOPCHOP program (version v3). Each of the full-length coding sequences of SpCas9-HF1-ssrA and FnCpf1 was globally shuffled to eliminate the TSD motifs of IS1 (IS1-8: NRAWWWWN), IS5 (IS5: YTAR), and IS10 (IS10: NRCWNWRYN), together with all repeats greater than or equal to 10 nucleotides using the software “Gene Designer” (version 2.0). Alignments of the sequencing results were performed using the algorithm MUSCLE (version v5) with the default settings, and subsequently displayed with the R-package “ggmsa” (version v1.4.0). A local BLASTn (version 2.12.0+) search of ISs against CRISPR-Cas gene clusters was performed with a cut-off of 100% sequence identity and 100% coverage. The graphical representations of candidate CRISPR-Cas gene clusters were visualized using the R package gggenes (version 0.4.1). We also employed a more sensitive software pipeline, ISEScan (version v1.7.2.1) to enable a more comprehensive detection of ISs transpositions into cas genes. We analyzed the presence of MITEs within cas genes of the CRISPRCasdb database using MITE-Tracker pipeline (no assigned version). Spacers of the CRISPR loci containing IS insertions into cas genes were predicted by CRISPRCasFinder (version 4.3.2). Identification of ORFs and initial annotations were rapidly conducted with the “Prokka” annotation pipeline (version 1.14.6). Using the resulting *.faa format file as input, antimicrobial resistance genes were detected by ResFinder (version 4.0). Taxonomy lineage for each identified genomic sequence was retrieved from the NCBI taxonomy database using TaxonKit tool (version 0.10.1). The fraction of genomes harboring each IS family in each taxonomic category was calculated and visualized by R package “pheatmap” (version 1.0.12). The total number of IS families per genome was computed for each taxonomic category and then plotted in boxplot format by the “ggplot2” package (version 3.4.2). The flanking regions spanning 8 bp and 9 bp for IS1 and 9 bp for IS10, upstream and downstream of each specific genome sequence, were extracted by Seqkit tool (version 2.2.0). The sequences immediately adjacent to the

element boundaries were detected by the pattern search program PatScan (no assigned version). Bioconductor R package "motifStack" (version 1.44.1) was used to generate the motif logo. A comparative analysis of IS transposition spectrum diversity across various genes was conducted and plotted using OncoPrint within the Bioconductor R package "ComplexHeatmap" (version 2.16.0). We used DefenseFinder (version 1.0.2) to search for prokaryotic antiviral systems. All data analysis and statistic were done in R 4.2.2 using RStudio. Both of the GraphPad Prism (version 9.5.1) and RStudio were used to plot figures. The graphs were then modified in Adobe Illustrator (version 26.1).

For manuscripts utilizing custom algorithms or software that are central to the research but not yet described in published literature, software must be made available to editors and reviewers. We strongly encourage code deposition in a community repository (e.g. GitHub). See the Nature Portfolio guidelines for submitting code & software for further information.

Data

Policy information about availability of data

All manuscripts must include a data availability statement. This statement should provide the following information, where applicable:

- Accession codes, unique identifiers, or web links for publicly available datasets
- A description of any restrictions on data availability
- For clinical datasets or third party data, please ensure that the statement adheres to our policy

All relevant data are included in the paper and/or its supplementary information files. Source data are provided as a Source Data file. All whole genome sequencing data of CRISPR-tolerant mutants were deposited in the National Center for Biotechnology Information (NCBI) Sequence Read Archive (SRA) repository under project number PRJNA884016, and the assembled chromosome sequences were also submitted to the GeneBank with accession numbers given in Table S6. Source data are provided with this paper.

Research involving human participants, their data, or biological material

Policy information about studies with human participants or human data. See also policy information about sex, gender (identity/presentation), and sexual orientation and race, ethnicity and racism.

Reporting on sex and gender	N/A.
Reporting on race, ethnicity, or other socially relevant groupings	N/A.
Population characteristics	N/A.
Recruitment	N/A.
Ethics oversight	N/A.

Note that full information on the approval of the study protocol must also be provided in the manuscript.

Field-specific reporting

Please select the one below that is the best fit for your research. If you are not sure, read the appropriate sections before making your selection.

☒ Life sciences ☐ Behavioural & social sciences ☐ Ecological, evolutionary & environmental sciences

For a reference copy of the document with all sections, see nature.com/documents/nr-reporting-summary-flat.pdf

Life sciences study design

All studies must disclose on these points even when the disclosure is negative.

Sample size	Sample sizes are indicated for each experiment and were chosen based on similar studies. The electro-transformation assays were performed in triplicate. For the qPCR assays, three independent biological replicates were included.
Data exclusions	No data was excluded.
Replication	All transformation assays were replicated for at least two times and the validity of the experiments was successfully reproduced in each case.
Randomization	Because there were no need to allocate samples into experimental groups in essence, this is not relevant to our study.
Blinding	Because there were no need to allocate samples into experimental groups in essence, this is not relevant to our study.

Reporting for specific materials, systems and methods

We require information from authors about some types of materials, experimental systems and methods used in many studies. Here, indicate whether each material, system or method listed is relevant to your study. If you are not sure if a list item applies to your research, read the appropriate section before selecting a response.

Materials & experimental systems

n/a	Involved in the study
☒	☐ Antibodies
☒	☐ Eukaryotic cell lines
☒	☐ Palaeontology and archaeology
☒	☐ Animals and other organisms
☒	☐ Clinical data
☒	☐ Dual use research of concern
☒	☐ Plants

Methods

n/a	Involved in the study
☒	☐ ChIP-seq
☒	☐ Flow cytometry
☒	☐ MRI-based neuroimaging
